# Exploring Human Metabolome after Wine Intake—A Review

**DOI:** 10.3390/molecules28227616

**Published:** 2023-11-15

**Authors:** Pelagia Lekka, Elizabeth Fragopoulou, Antonia Terpou, Marilena Dasenaki

**Affiliations:** 1 Food Chemistry Laboratory, Department of Chemistry, National and Kapodistrian University of Athens, Panepistimiopolis Zographou, 15771 Athens, Greece; lekkap@chem.uoa.gr; 2 School of Health Science and Education, Department of Nutrition and Dietetics, Harokopio University, 17671 Athens, Greece; efragop@hua.gr; 3 Department of Agricultural Development, Agrofood and Management of Natural Resources, School of Agricultural Development, Nutrition & Sustainability, National and Kapodistrian University of Athens, 34400 Psachna, Greece; aterpou@agro.uoa.gr

**Keywords:** metabolomic studies, wine consumption, biological samples, target and non-target analysis, diet biomarkers

## Abstract

Wine has a rich history dating back to 2200 BC, originally recognized for its medicinal properties. Today, with the aid of advanced technologies like metabolomics and sophisticated analytical techniques, we have gained remarkable insights into the molecular-level changes induced by wine consumption in the human organism. This review embarks on a comprehensive exploration of the alterations in human metabolome associated with wine consumption. A great number of 51 studies from the last 25 years were reviewed; these studies systematically investigated shifts in metabolic profiles within blood, urine, and feces samples, encompassing both short-term and long-term studies of the consumption of wine and wine derivatives. Significant metabolic alterations were observed in a wide variety of metabolites belonging to different compound classes, such as phenolic compounds, lipids, organic acids, and amino acids, among others. Within these classes, both endogenous metabolites as well as diet-related metabolites that exhibited up-regulation or down-regulation following wine consumption were included. The up-regulation of short-chain fatty acids and the down-regulation of sphingomyelins after wine intake, as well as the up-regulation of gut microbial fermentation metabolites like vanillic and syringic acid are some of the most important findings reported in the reviewed literature. Our results confirm the intact passage of certain wine compounds, such as tartaric acid and other wine acids, to the human organism. In an era where the health effects of wine consumption are of growing interest, this review offers a holistic perspective on the metabolic underpinnings of this centuries-old tradition.

## 1. Introduction

**Figure 1:**
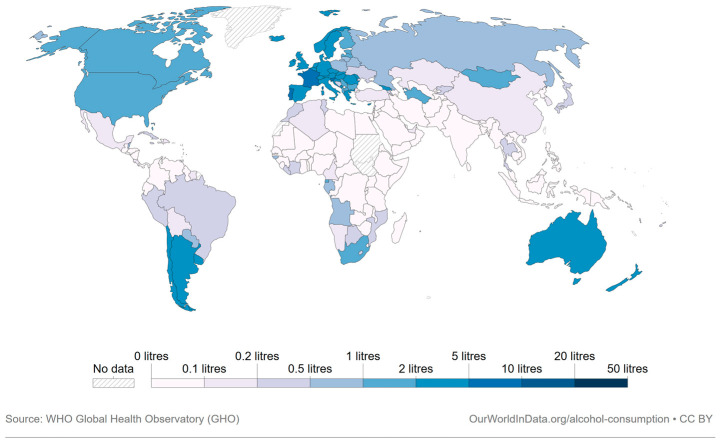
Per capita wine consumption in 2019, quantified in liters of absolute alcohol per annum (approximately 0.12 L of pure alcohol are found in 1 L of wine) [1].

According to the International Organization of Vine and Wine (OIV), global wine consumption has been steadily increasing over the years. In 2019, the estimated global wine consumption reached 244 million hectoliters, as shown by country in Figure 1, indicating its continued popularity worldwide [1]. 

**Figure 2:**
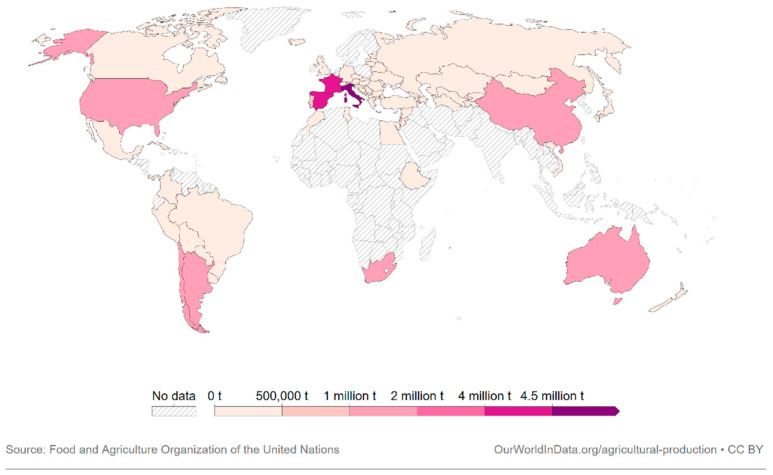
Wine production measured in tonnes by country, 2020 [2].

Wine is produced and enjoyed in numerous countries around the world, including traditional wine-producing regions like France, Italy, Spain, and new-world producers such as the United States, Argentina, Australia, and Chile, as depicted in Figure 2. The widespread cultivation and consumption of wine illustrate its global popularity [2].

Nevertheless, wine’s popularity and significance extend far beyond the present day. Its therapeutic use can be traced back to 2200 BC, making it the oldest known medicine [3]. In the early 1990s, the French Paradox sparked an intense scientific investigation into the cause of lower death rates in France than in the USA, in spite of comparable plasma cholesterol levels and other similar risk factors. This inquiry revealed a noteworthy finding: moderate regular wine intake (1–2 glasses per day) has been linked to lower cardiovascular mortality and risk of heart disease [4]. These advantages have been related to improved antioxidant capacity, lipid profile alterations, and anti-inflammatory actions. Following the initial link between the French Paradox and red wine consumption, groundbreaking research emerged [5,6,7]. A comprehensive review study, with the aim to map the scientific research on wine and health, was conducted in 2013 by Aleixandre et al. and they came up with more than 400 papers published on this topic in the most productive journals between 2002–2011 [8]. However, the pursuit of knowledge in this field continues unabated. In 2011, Contstanzo et al. conducted a comprehensive meta-analysis regarding fatal and non-fatal cardiovascular events, wherein they identified a J-shaped relationship between elevated consumption of wine and beer and vascular risk [9]. Moreover, in the year 2023, Lucerón-Lucas-Torres et al. complete a meta-analysis in the association between wine consumption with cardiovascular disease and cardiovascular mortality. Their findings unveiled an inverse correlation between wine consumption and cardiovascular mortality, cardiovascular disease (CVD), and coronary heart disease (CHD) [10]. Similar results were presented also by Lombardo et al. (2023) were the beneficial effect of wine consumption on antioxidant levels, markers related to thrombosis and inflammation, lipid profile, and improvements in the composition of gut microbiota, was reported [11]. It is noteworthy to mention that, to the best of our knowledge, a review study regarding the metabolic alterations caused in the human organism due to wine consumption has not been published before. 

To comprehensively explore the health benefits associated with wine consumption, it is crucial to first understand its complex chemical composition. Wine is a natural beverage derived from the direct fermentation of grapes and it consists of compounds originating from grape berries, yeast, bacteria and oak, all of which contribute to the unique chemical profile and health advantages of wine. Wine contains numerous components such as water, ethanol, organic acids glycerol, sugars, certain amino acids, volatile compounds, and polyphenolic compounds, among others [12,13]. Hence, it is of utmost importance to comprehend the mechanisms by which these various classes of wine compounds can interact with the human body. The interpretation of wine compounds metabolism and metabolic pathways in the human body after wine consumption has been in the spotlight of research in the last years, with the application of “-omics” technologies playing an invaluable role in this upturn [12].

“Omics” refers to scientific branches that encompass the measurement and analysis of different disciplines, such as metabolomics (metabolites) including lipidomics (lipids), proteomics (proteins), and genomics (genes). The term “Omics” originates from the Latin suffix “ome”, meaning many [14]. In this current review, we chose to focus on metabolomics due to its increasing use in recent years for detecting food-based metabolites in human biological samples [15]. 

Metabolomics, which is the study of small molecules or metabolites, particularly those with a molecular weight below 1500 Da in biological samples [16,17], stands out as an advantageous technique compared to other “omics” approaches. Metabolites, including among other carbohydrates, amino acids, lipids, energy metabolites, vitamins, co-factors, nucleotides, and xenobiotics, could be influenced from the diet intake, environmental exposure, microbial metabolism, and pharmacotherapy [18]. Analyzing how metabolites respond to stimuli provides a snapshot of an organism’s metabolism, akin to a molecular fingerprint, effectively reflecting the organism’s overall biological condition [19]. This approach is being increasingly utilized in various areas of study, including the analysis of food components, the identification of diet-derived metabolites, assessment of their bioavailability and metabolism, examination of metabolic activities in the gut microbiota, and understanding the physiological response to specific dietary patterns, foods, or nutraceuticals [20].

This comprehensive review critically examines a wide range of metabolomic studies that have focused on investigating the changes in metabolic profiles following both short-term and long-term wine consumption. All these 51 studies included in our review present the investigation of human metabolome of both healthy and unhealthy participants that consumed either wine, dealcoholized wine, or wine extract in capsule form. Biological samples were subjected to target and/or nontarget analysis using liquid chromatography and gas chromatography coupled with mass spectrometry, or H-NMR spectroscopy techniques. By meticulously analyzing and synthesizing the findings from these studies, we aim to provide a comprehensive and detailed understanding of the metabolic alterations that occur as a result of wine consumption. These alterations are observable across a range of compound classes, spanning from endogenous metabolic substances to wine compounds. For better understanding, we have compiled tables listing all the distinct compound classes that demonstrated significant differences compared to the samples taken prior to wine consumption or compared to control group. These tables could present an invaluable tool for the development of ready-to-use databases enabling the non-target/suspect screening of wine-related metabolites in biological samples. 

## 2. Clinical Studies

Several clinical investigations have been conducted to assess the influence of wine consumption on the human metabolome. These studies can be classified into two main categories: interventional studies and observational studies [21]. Findings from these investigations have been consolidated and are presented comprehensively in Table 1 and Table 2. Furthermore, interventional studies were categorized into: parallel, crossover and randomized studies. Understanding the distinction between these types is essential in interpreting the findings and implications of the research. In parallel studies, different groups of subjects are assigned to distinct interventions, allowing for between-group comparisons [22,23,24,25,26,27,28,29,30,31]. On the other hand, crossover studies enable each subject to serve as their own control by undergoing both interventions in a sequential manner [32,33]. And lastly randomized controlled trials are randomly allocated to either the treatment or control arms [22,23,34]. In Supplementary Table S1, a comprehensive compilation of information regarding interventional and observational clinical studies in the field of wine metabolomics research is presented.

### 2.1. Interventional Studies

#### 2.1.1. Wash out Period

The significant majority (more than 70%) of the clinical studies concerning wine consumption were interventional studies. In these studies, before the intervention, the participants underwent a washout period during which they refrained from consuming polyphenol-rich foods, as well as wine and other alcoholic beverages (see Table 1). Following the washout period, the subjects entered an intervention period that lasted from a day or a specific number of 5–20 days or 3–8 weeks. During the intervention period, the participants were given explicit instructions to consume a predetermined volume of a particular wine daily. Alternatively, in some studies, participants were administered dealcoholized wine [31,32,35,44,45,47,51,54] or wine derivatives [36,49,50,51,52,55,56]. It is important to note that throughout the interventional studies, participants were instructed to maintain their regular diet and abstain from consuming other alcoholic beverages. Notably, among the selected studies, only the investigation conducted by Vitaglione et al. (2005) specifically explored the potential relationship between the type of meal consumed and the variety of red wine consumed by investigating resveratrol metabolites bioavailability [30]. 

#### 2.1.2. Duration of the Study

The duration of interventional studies ranged from one day to 8 weeks. Specifically, as it can be seen in Table 1, fifteen studies had a duration of only one day, while three studies extended their interventions over 5 to 20 days. The majority of the studies encompassed a timeframe of 3 to 4 weeks. Notably, one study stood out with a substantial duration of 8 weeks [71]. These variations in study duration highlight the diverse timeframes employed to examine the intended interventions.

#### 2.1.3. Participants

The number of participants in a clinical study is crucial for ensuring statistical power and drawing meaningful conclusions. A large participant pool enhances the study’s ability to detect effects or differences accurately. However, finding a diverse group of participants that meet the study’s criteria is a time-consuming and resource-intensive task. As a result, most interventional clinical studies concerning wine consumption had a range of 5 to 61 participants (Table 1). Notably, more than half of these studies deliberately included both male and female participants, thereby achieving a balanced gender representation. In contrast, 14 out of 37 studies specifically recruited only male subjects. This decision was made to minimize potential confounding factors related to menstrual cycle phase variability in females, which could impact processes such as wine absorption, metabolism, and excretion [41]. For instance, in the study conducted by Haas et al. (2022), the exclusive inclusion of male participants aimed to achieve sample homogeneity, considering potential differences in alcohol metabolism and trimethylamine-N-oxide (TMAO) metabolism between sexes [34].

Besides sex, also the health of participants plays a pivotal role in clinical studies due to its multifaceted significance. The majority of interventional studies in this review centered on healthy participants, affording valuable insights into the impact of wine consumption on individuals without pre-existing health conditions. Additionally, 5 studies focused on participants with type 2 diabetes (T2D) or those exhibiting at least three coronary heart disease (CHD) risk factors, or cardiovascular risk factors (CVRFs) shedding light on the potential benefits or risks of wine consumption for individuals with these conditions [44,45,47,48,54]. It is worth noting that 3 studies within this review did not specify the health status of their participants [33,56,57], while an additional 3 studies specifically focused on participants with mild hypertension [36,49,50]. Additionally, one study explored the impact of wine consumption on individuals diagnosed with coronary artery disease (CAD) [34].

#### 2.1.4. Type and Amount of Wine/Wine Derivatives

The choice of wine variety consumed significantly influences the resulting impact on human metabolome [61]. Within the interventional studies included in this review, it was customary for participants to consume a specific type of red, white, sparkling or dealcoholized wine as part of the study protocol. A small number of studies employed the consumption of wine extracts either as additives in wine or as individual supplements. The various types of interventions are presented in Table 1.

The interventional studies encompassed a range of red wine varieties, including Merlot, Pinot Noir, Cabernet Sauvignon, Cabernet Franc, Lemberger, Shiraz, as well as Spanish variety such as Tempranillo, and Italian varieties such as Lambrusco and Aglianico. These specific red grape varieties were purposefully selected based on their notable phenolic content [37,48] with some studies reporting also the phenolic profile of the utilized wines [46,47,48,54]. In addition to regular red wines, a significant number of studies examined dealcoholized red wines. Moreover, although the majority of studies focused on regular red wine, three published papers explored white wine and only one study investigated sparkling wine [58]. 

The typical daily wine intake in the studies ranged from 200 to 272 mL. The selection of a 250 mL dosage was based on evidence indicating that this quantity of red wine is associated with beneficial effects [34]. However, there were three studies that provided a lower amount of 120 mL [32,33,39], while two one-day interventional studies deviated from the norm by administering a higher volume of 500 and 600 mL of wine [30,43]. 

### 2.2. Observational Studies

#### 2.2.1. Food Frequency Questionnaires (FFQs)

Fourteen metabolomic studies investigating the human metabolome after wine intake were observational studies. In the field of observational studies, traditional approaches to assessing regular eating habits have relied on self-reported tools to evaluate diets, such as food frequency questionnaires (FFQs). Consequently, many studies investigating wine consumption use these self-reported measures as a means of data collection. However, it is important to acknowledge that self-reported measures are inherently prone to both random and systematic measurement errors, which may compromise the accuracy and reliability of the findings [59]. Recognizing these limitations, Regal et al. (2017) acknowledge the challenges associated with accurately recalling food/beverage consumption, especially concerning wine intake, due to social preconceptions surrounding alcoholic beverages. To address these challenges, they aimed to investigate the potential of resveratrol as a reliable biomarker for objectively measuring wine consumption within a specific population. The study concluded that resveratrol can serve as a valuable dietary biomarker only when used in conjunction to dietary tools since this stilbene is naturally present also in several plant species [61]. 

#### 2.2.2. Participants

Observational studies, which rely on passive observation rather than active interventions, usually include a broader range of subjects. In the studies presented in this review the participant numbers range from 222 to 3559 individuals [63,65] with the exception of a single study featuring only 25 participants [61]. More than half of the studies ensured representation from both sexes by incorporating both male and female subjects as participants. However, two specific clinical studies deviated from this approach and focused exclusively on female participants. One study examined female twins [63] while the other focused on women experiencing menopause [72].

Apart from gender, health status of the participants is of utmost importance. Among the observational studies reviewed seven chose to recruit participants from the general population [59,63,64,66,67,68,70], while 3 studies specifically focused on healthy participants. Additionally, 3 studies focused on participants with T2D, CHD risk factors and CVRFs, shedding light on the potential benefits or risks of wine consumption for individuals with these conditions [12,60,71]. Lastly, Playdon et al. (2016) included participants diagnosed with new or recurrent cases of colorectal adenoma [69].

## 3. Biological Samples, Analytical Techniques and Statistical Analysis

The process of sample preparation and analysis plays a pivotal role as it directly influences the quality and reliability of the study results. Table 1 and Table 2 provide a comprehensive overview of the various protocols of sample preparation, analytical techniques and statistical analysis utilized in metabolomic studies related to wine consumption. Statistical analysis emerged as a prevalent approach in the majority of these studies, facilitating the elucidation of significant changes and trends in the metabolome.

### 3.1. Biological Samples Preparation

**Figure 3:**
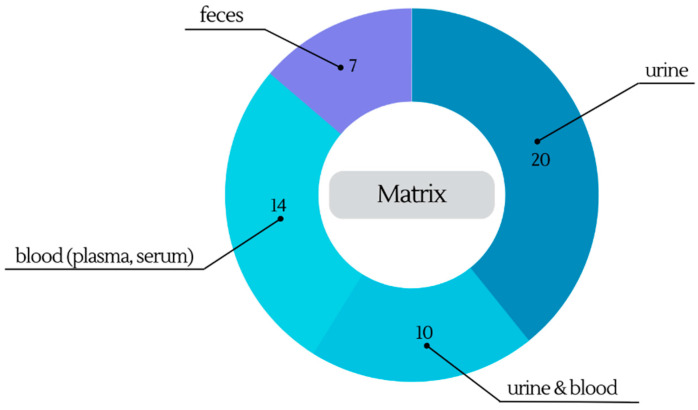
Distribution of collected biological samples in clinical studies of wine metabolism.

In metabolomic research, the choice of biological samples is pivotal to unraveling the intricate web of metabolic processes. Figure 3 illustrates the distribution of commonly collected biological samples, highlighting the prominence of urine, blood, and feces in metabolomic investigations. 

#### 3.1.1. Urine 

Urine, one of the extensively examined biofluids in metabolomic research, holds great significance due to its ease of collection and its lower complexity. Besides its convenience, urine plays a crucial role as an excretory pathway for water-soluble metabolites and xenobiotics in the body. Consequently, the analysis of the urinary metabolome holds significant potential in providing valuable insights into diseases and dietary intake [16,73]. As a result, as you can see in Table 1 and Table 2, almost half of the metabolomic studies focused exclusively on investigating the urine metabolome after wine intake while an additional 10 studies explored both urine and blood samples for a more comprehensive study. 

The two most popular sample preparation techniques for urine prior to chromatographic analysis were liquid-liquid extraction (LLE) with ethyl acetate or acetonitrile (ACN) and solid phase extraction (SPE). Additionally, in some studies, researchers performed enzymatic hydrolysis before extraction with the purpose of exclusively examining polyphenol aglycones [67,68]. Lastly, some research groups preferred to carry out minimum sample preparation before analysis and chose the dilute-and-shoot [41,42,65,70] or direct injection method [24,25,69]. In addition, a subset of studies that conducted gas chromatography (GC) analysis incorporated an additional step of derivatization. Among these studies, the preferred derivatization reagent was bis(trimethylsilyl)trifluoroacetamide (BSTFA), while others utilized mixtures containing BSTFA or N-methyl-N-(trimethylsilyl)trifluoroacetamide (MSTFA), in combination with trimethylchlorosilane (TMCS).

Regarding sample preparation for proton nuclear magnetic resonance analysis (H-NMR), urine exhibits a notable pH range, typically ranging from 5 to 8, and this variance significantly influences the observed chemical shifts in NMR spectra. To effectively address this challenge, a phosphate buffer with a pH of 7.4 is routinely incorporated into the sample preparation process [74,75]. This buffer ensures pH stability, minimizing variations across different urine samples and enhancing the overall consistency and reliability of the analysis. Additionally, sodium azide is added to maintain sample integrity by effectively controlling bacterial growth. 

#### 3.1.2. Blood (Plasma, Serum)

Blood serum and plasma are widely utilized biofluids in metabolomic studies, ranking second in frequency after urine. The choice between serum and plasma as the preferred biofluid for metabolomic analysis remains an important and unresolved question, with different research groups opting for either serum or plasma [73]. Within the studies presented in the current review, 14 studies exclusively investigated the blood metabolome after wine intake, with 11 studies utilizing plasma [29,33,34,37,38,39,40,59,60,61,62], 2 employing serum [30,64] and Pallister et al. (2016) investigating both serum and plasma [63]. Among the studies that explored both urine and blood metabolome, 7 of them involved plasma [31,51,52,53,54,55,56] and 3 serum samples [57,58,72]. Consequently, the prevailing trend among researchers indicates a preference for plasma samples over serum samples [57,58,72].

A significant obstacle encountered in the analysis of metabolites in intact serum/plasma is the substantial interference caused by the presence of a large quantity of serum/plasma proteins (6–8 g/dL) [75]. Urpi-Sarda et al. (2005) [37] and Gonzalez et al. (2020) [40] conducted comprehensive studies investigating optimal sample preparation techniques for plasma according to the extraction recovery of resveratrol and other food metabolites, following moderate wine consumption [40]. Both studies independently found that protein precipitation (PPT) was the most effective sample preparation when compared to liquid-liquid extraction (LLE) and solid phase extraction (SPE) methods, which are the three most commonly used blood sample preparation techniques. Consistent with the approach taken for urine sample preparation, some researchers also apply enzymatic hydrolysis prior to implementing any extraction technique in plasma samples [29,33,38,39].

In studies employing gas chromatography (GC) for sample analysis, an additional derivatization step was implemented. The preferred derivatization reagent utilized by these studies was BSTFA. Furthermore, alternative derivatization mixtures comprising BSTFA in conjunction with TMCS [56], o-methylhydroxylamine, or pure MSTFA were employed by other researchers [52,55]. In the current body of research, blood samples have not been employed for H-NMR analysis.

#### 3.1.3. Feces

Feces are a valuable addition for assessing the combined pool of endogenous and microbial metabolites, often referred to as the hyperbolome [76]. 

In relation to the preparation of fecal samples, the methodologies implemented in the literature predominantly revolved around the dissolution of fecal matter to form a fecal solution. Subsequently, the fecal solution was subjected to extraction using acetonitrile (ACN) [23,26,28,35]. Notably, only one study deviated from this approach and employed solid-phase microextraction (SPME) as an alternative technique for sample preparation [22]. When it comes to the preparation of fecal water samples for H-NMR analysis, Jacobs et al. (2008) was the sole study that employed this analytical technique [36]. In their approach, the fecal water samples were first alkalized using sodium hydroxide (NaOH), followed by acidification with formic acid. Furthermore, TSP was used as an internal standard for metabolite quantification.

### 3.2. Analytical Techniques

**Figure 4:**
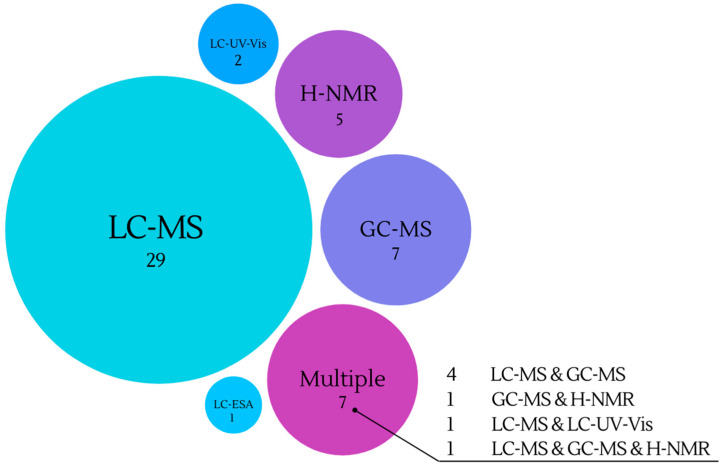
Analytical techniques employed in clinical studies of wine metabolism.

It is evident that numerous analytical methods can be applied to investigate the human metabolome following wine consumption, thus raising the question of which particular method from the ‘metabolomics toolbox’ should be selected. As illustrated in Figure 4, LC-MS, GC-MS, and H-NMR were frequently chosen. Additionally, many studies adopted a multi-faceted approach, combining multiple techniques within the same investigation.

#### 3.2.1. LC-MS 

Liquid chromatography (LC) is a widely employed separation technique, particularly for complex samples such as biofluids. It offers advantages like simplified sample preparation and shorter run times compared to gas chromatography (GC). The versatility of LC stems from the availability of various stationary phases and the ability to use different mobile phases, allowing for the analysis of a broad range of compounds, from moderately to highly polar substances, as well as low volatility and thermolabile compounds [17]. Moreover, the coupling of LC with Mass Spectrometry (MS) offers unparalleled detection and identification capabilities, which allows not only the determination of well-known metabolites with remarkable sensitivity and specificity, but also the untargeted study of unknown wine metabolites and related compounds [77,78]. 

Reverse phase liquid chromatography (RPLC) is most commonly used in metabolomics to separate polar metabolites and non-polar lipids. However, it may not retain highly polar or ionic compounds effectively. In such cases, hydrophilic interaction liquid chromatography (HILIC) complements RPLC by retaining polar or ionic metabolites, including compounds related to key metabolic pathways [16,79,80]. The choice of a suitable stationary phase can be tailored to the specific compounds of interest, especially in targeted metabolomics. For untargeted metabolomics applications, using multiple column types is essential [81]. In this review, most LC-based metabolomic analyses used RPLC, mainly C18 columns, with two studies using C8 columns [25,26]. One study employed HILIC columns for overall analysis, while another used them specifically for measuring trimethylamine N-oxide (TMAO) [34,60]. 

Mass spectrometry has been the primary platform for metabolomics applications because of its excellent sensitivity, selectivity, and remarkable compound annotation capabilities [16,17,82]. Therefore, it is not surprising that the vast majority of the wine intake metabolomic studies used LC-MS analysis for the determination of both targeted and untargeted metabolites.

Various ionization techniques, such electrospray ionization (ESI), electron impact (EI), and others, are used in metabolomic studies [83,84]. In most cases negative ionization was used, in order to determine phenolic metabolites; nevertheless recent studies utilize both positive and negative polarities [34,40,59,72].

Both low and high resolution mass analyzers have been used, with triple quadrupole mass analyzer being the predominantly employed [31,46,48,58,71]. However, most recent applications also employ the use of TOF [25,26,27], QTOF [24,70], [25,26,27] Orbitrap [34,60] and Fourier transform ion cyclotron resonance mass analyzers (FT-ICR-MS) [62,72]. In the majority of metabolomic studies target analysis was performed being the approach that focuses on identifying and quantifying selected metabolites [78,85]. On the other hand, non-target analysis allows the simultaneous determination of numerous unspecified metabolites giving the opportunity to compare metabolic profiles and track shifts in response to certain internal or external variables using high-resolution mass spectrometry (HRMS) [85,86].

To perform untargeted metabolomics using mass spectrometry, different MS acquisition techniques have been proposed: full scan, Data Independent acquisition (DIA), or Data Dependent acquisition (DDA) [87]. Both DDA and DIA have been used to address different questions: DDA relies on intensity-dependent precursor ion selection but potentially missing low-abundance metabolites in MS^2^ analysis, while DIA theoretically captures MS^2^ spectra for all precursor ions, enhancing low-concentration metabolite detection, but interpreting complex spectra poses informatics challenges [34,86]. In a characteristic untargeted metabolomic study, Haas et al. (2022) used DDA to investigate the effects of red wine consumption on the plasma metabolome [34].

#### 3.2.2. GC-MS 

Gas chromatography-mass spectrometry (GC-MS) serves as a widely employed platform in metabolomics. However, one significant practical limitation is that many classes of metabolites, such as phenolic compounds, sugars, nucleosides, amino acids, among others, cannot be directly analyzed due to their polarity and lack of volatility, requiring the precedence of a derivatization step [73] This derivatization process is time-consuming, has limited throughput and carries a risk of errors, thus introducing variability and the possibility of artifacts [88]. Additionally, GC-MS has a restricted mass range, and the molecular ion is frequently not detected due to fragmentation, which poses challenges for the identification of unknown compounds [79]. Nonetheless, GC-MS, particularly GCGC-MS, offers notable benefits, including exceptional resolution, high sensitivity, and robustness [88]. 

In metabolomic studies after wine intake it is evident that gas chromatography (GC) emerges as the second most frequently employed analytical technique. Out of the 51 studies reviewed, a total of 13 studies opted for GC as the method of choice to identify the human metabolome following both long-term and short-term wine consumption (Table 1 and Table 2). The majority of these studies used columns constituted of 5% phenyldimethylpolysiloxane in conjunction with polyethylene glycol (PEG), while some utilized columns consisting of 50% phenyl and 50% dimethylpolysiloxane. Additionally, one study employed a column based on polyvinylpolypyrrolidone (PVPP). Researchers primarily focused on the analysis of phenolics, lipids, and organic acids using GC-MS. Phenolic compounds, including 4-hydroxymandelic acid [49,56], pyrogallol [49,55], vanillic and its derivatives, as well as numerous flavonoids as elucidated by Donovan et al. (1999, 2002) have been extensively investigated [32,33]. Moreover, recent work by Belda et al. in 2021 has expanded the scope to include the analysis of short-chain fatty acids and medium-chain fatty acids using SPME-GCMS [22].

#### 3.2.3. NMR

H-NMR spectroscopy offers several advantages in the field of metabolomics. Firstly, H-NMR is highly reproducible and provides quantitative measurements, ensuring reliable analysis of metabolites. H-NMR offers the advantage of absolute quantification, with signal intensities directly proportional to the concentration of nuclei in the sample. Additionally, it allows for the analysis of intact biological samples without the need for extensive sample preparation, reducing the potential for analytical variability. H-NMR also enables the identification of unknown metabolites, making it valuable for comprehensive profiling of complex metabolic mixtures. Another advantage is its non-destructive nature, which preserves the sample for further analysis or retesting. Furthermore, H-NMR facilitates the study of metabolic pathways and fluxes by utilizing stable isotope labeling techniques. With its ability to detect multiple atomic nuclei, including 1H, H-NMR offers flexibility in metabolite detection. Overall, these advantages make H-NMR spectroscopy a powerful and versatile tool for investigating metabolites and their role in biological systems [74,75,89].

In contrast to the aforementioned benefits, it is noteworthy that out of the 51 studies examined, a mere seven studies opted to utilize H-NMR as their analytical approach. Among these, two studies employed H-NMR specifically for target analysis of hippuric acid and SCFAs [36,55]. This choice was motivated by the fact that hippuric acid stands out as the most abundant phenolic acid in urine, and its concentrations often tend to be underestimated when employing MS detection due to saturation effects. The remaining five studies employed nontarget analysis with the aim of assessing the effects of moderate wine, dealcoholized wine, or wine derivatives following four weeks of intake [44,45,47,49,50]. In the metabolomic applications included in this review all H-NMR samples presented with internal standard TSP and most of them used sodium azide and phosphate buffer [44,45,47].

Studies using H-NMR tend to identify fewer statistically significant metabolite differences compared to those employing LC-MS or GC-MS techniques. For example, in H-NMR analysis, only the study by Van Dorsten (2010) detected 19 metabolites with significant changes before and after 4 weeks of a wine derivative consumption [49]. In contrast, the remaining seven metabolomic studies utilizing H-NMR determined only 1 to 11 compounds. Additionally, H-NMR analysis is predominantly used for the determination of smaller molecular weight compounds, particularly ethanol, amino acids and their derivatives like threonine and alanine [44,47,50,55]. Notably, only five phenolic compounds were detected to have statistical difference before and after wine consumption using H-NMR (3-hydroxyphenylpropionic acid, vanilmandelic acid, dihydroferulic acid, isovanillic acid, 3,4-dihydrophenylglycol) while 116 were detected with LC-MS.

### 3.3. Chemometrics

Statistical analysis plays a crucial and indispensable role in metabolomic studies, as it enables the identification of statistically significant metabolomic changes, the elucidation of potential biomarkers [25], and the exploration of intricate metabolic pathways influenced.

**Figure 5:**
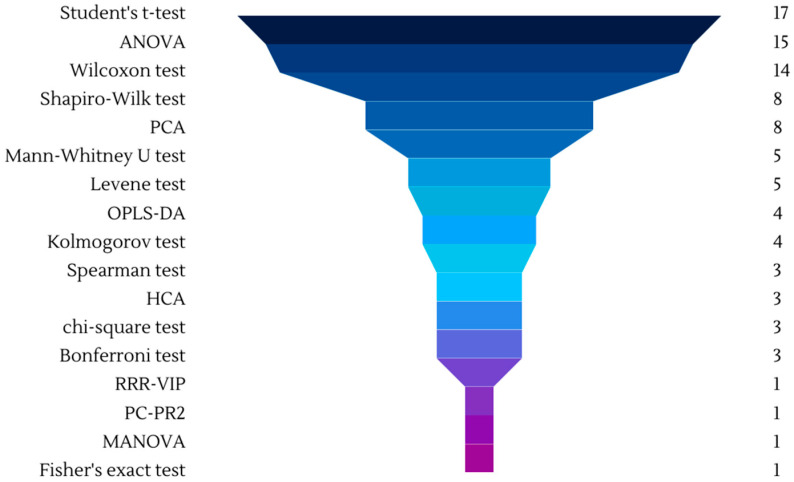
Statistical analysis employed in clinical studies of wine metabolism.

The statistical analysis employed in the reviewed studies revealed a diverse range of techniques used to assess the impact of wine consumption on metabolomic profiles as can be seen in Figure 5.

The most frequently utilized parametric tests included Student’s *t*-test and ANOVA, which examined mean differences and evaluated metabolite level variations among multiple groups or conditions (Table 1 and Table 2). ANOVA was used in many different types of studies, for example Urpi-Sarda et al. (2015) used it to compare changes in phenolic metabolites in plasma and urine after wine intervention treatments, while Motilva et al. used ANOVA to exclude extreme outliers of metabolites after a 1-day of dealcoholized wine intake and wine derivatives intake. Non-parametric tests such as the Wilcoxon test [34,41,48,55] and Mann–Whitney U [41,45,55,62] test were employed to examine changes in paired and independent samples, respectively. Regueiro et al. (2014) used these tests to separate group 1 with 100 mL of wine intake, with group 2 with 200 mL of intake and group 3 with 300 mL of intake [41]. Additional statistical techniques included the Shaprio-Wilk test (in 8 studies) to assess data normality assumptions, the Kolmogorov test (in 4 studies) to examine metabolite distribution characteristics and the Spearman test [34,66] to explore correlations between metabolites (Table 1 and Table 2). 

Multivariate analysis techniques such as OPLS-DA [24,25,56,70] and MANOVA [29] were utilized to identify patterns, discriminant features, and joint effects of multiple variables. Other techniques included the Levene test [22,58], LASSO regression [62,67,69], chi-square test [62,64,69] and hierarchical cluster analysis (HCA) to address variance equality [24,54,70], feature selection, categorical associations, and clustering of metabolites, respectively. For example, HCA was used in Urpi-Sarda (2015) to cluster metabolites between the interventions with gin and wine [54] while OPLS-DA was employed to detect statistical differences before and after wine consumption [25]. Edmands et al. (2015) employed OPLS-DA for an observational study, aiming to identify biomarkers that met the intake criteria for various polyphenol-rich foods [70]. Other techniques employed in specific studies included Fisher’s exact test for FDR calculation [62,69], the Omnibus K2 D’Agostino-Pearson test for assessing normality [41], RRR-VIP for variable importance analysis [67], linear Support Vector Machine for classification of potential dietary biomarkers [72], the Clopper-Pearson exact binomial method for analyzing binary outcomes. 

Overall, the primary objective of any metabolomic study is to generate comprehensive metabolic profiles from both test and control samples, facilitating the identification of potential biomarkers. Regarding sample preparation, achieving comprehensive extraction of the extensive sample metabolome is an essential yet challenging prerequisite. Exploring the potential benefits of automation in this process holds promise. It is apparent that several analytical methods can be employed for this purpose, thereby raising the question of which specific method from the “metabolomics toolbox” should be given preference. Nevertheless, it is currently not possible to provide a straightforward answer to this query. Metabolomics analysis necessitates the integration of pragmatism, skepticism, and the effective utilization of available technology. Consequently, none of the individual analytical techniques outlined can independently yield a comprehensive profile due to inherent limitations in sensitivity or potential biases towards specific analytes. To overcome these challenges, a prudent strategy for achieving global metabolic profiling involves employing a diverse range of complementary analytical methods, there by maximizing coverage within a reasonable time frame [83,84,90]. 

## 4. Metabolites–Biomarkers of Wine Intake

**Figure 6:**
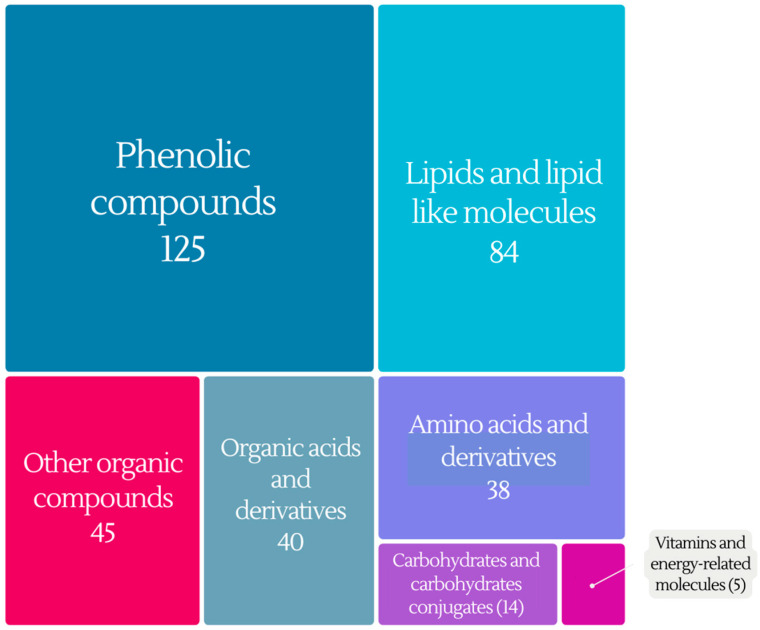
Categorization of metabolites affected by wine intake.

The present review paper offers a comprehensive documentation of the metabolites whose concentrations undergo significant changes after wine consumption, as monitored in biological samples after long-term, or short-term wine consumption. This extensive research yielded a list comprising approximately 600 compounds that demonstrate statistical differences before and after wine consumption. Rigorous processing and scrutiny of this data involved eliminating duplicate compounds that might appear with different names and excluding unknown entities that may have emerged from non-targeted analyses. The remaining 361 compounds were then classified into distinct categories based on their chemical structure: (i) phenolic compounds, (ii) lipids and lipid-like molecules, (iii) organic acids, (iv) amino acids and derivatives, (v) carbohydrates and carbohydrate conjugates, and (vi) organic compounds as it can be seen in Figure 6. This classification aligns with the preferred categorization used in The Human Metabolome Database, ensuring clarity and consistency in our analysis.

For the majority of these metabolites we were able to pinpoint their behavior, either up-regulating or down-regulating after wine intake; however for some metabolites this information was unknown, as it was only reported that they exhibited statistical significant differences. It is also important to note that all studies uniformly agree on the direction of change (increase or decrease) for the examined metabolites following wine consumption. 

### 4.1. Phenolic Compounds

Phenolic compounds emerged as the most abundant group of metabolites correlated to wine intake, comprising a remarkable total of 125 compounds. In Table 3 all phenolic metabolites that have been determined in biological samples after wine intake are presented. In addition to target screening applications, recent advancements in analytical techniques have facilitated the untargeted analysis of biological samples that can reveal the entire phenolic profile [22,24,34,59,64]. These metabolites belong to different phenolic compound classes, encompassing flavonoids, hydroxybenzoic acids, catechols, gallic acid metabolites, hydroxycinnamic metabolites, methoxyphenols, coumarins, phenylpropanoic acids and lignans.

In our comprehensive analysis, out of the 125 different phenolic compounds that were observed to vary significantly in the human metabolome after wine consumption, most of them were increased. Notably, within the phenolic compounds group, it’s important to emphasize that the categorization encompasses not only the parent compounds but also their glucosides and glucuronides forms. It is noteworthy that a substantial proportion of the phenolic compounds that exhibited an increase in concentration fall within the three categories of flavonoids, catechols, and gallic acid and metabolites. Conversely, the compounds displaying decreased concentrations predominantly belong to the hydrocinnamic acids, a category including caffeic acid sulfate, dihydrocaffeic acid 3-sulfate and piplartine.

Among the identified metabolites, resveratrol, vanillic acid, syringic acid, and (epi)catechin were the most characteristic compounds displaying statistical significantly differences after wine consumption. Resveratrol garnered remarkable attention as an essential component that brings to red wine’s health benefits [5]. Studies dating back to 2001 sought to unravel its pharmacokinetics and bioavailability [29,30,57,74], predominantly focusing on hydrolyzed samples of urine and blood. As analytical techniques advanced, researchers made significant strides in identifying various resveratrol metabolites, such as sulfates, glucuronides, and glucoside derivatives with a predominant presence in urine samples [40,48,66]. (Epi)catechin has also drawn attention. It has been under investigation since 1999 for its remarkable ability to offer protection against cardiovascular diseases (CVDs) [33,91]. In recent years, researchers have also identified its derivatives, particularly sulfates and methylated glucuronides [46,51].

Furthermore, a multitude of studies have documented the presence of gallic acid metabolites, encompassing gallic acid itself, methylgallic acids, methylgallic acid sulfates, and pyrogallol. Particularly intriguing observations have been made regarding 4-O-methylgallic acid, the primary metabolite of gallic acid [92], which has been detected in both urine and plasma of participants after wine intake [38,46,70]. Conversely, 3-O-methylgallic acid was exclusively identified in feces samples [26,28,35], thus revealing distinct distribution patterns between isomers within biological specimens. 

In addition to hydroxycinnamic acids, such as ferulic acid, which has been widely reported to possess diverse physiological functions [93,94], the detection of other significant metabolites after wine intake has been documented. Caffeic acid, known for its potential as a carcinogenic inhibitor [95], has been detected in both urine and plasma of participants after wine consumption. Furthermore, p-coumaric acid, displaying anti- inflammatory activities [96], has been identified in urine, plasma, and feces [35,46,54,76], in contrast to its isomer, m-coumaric acid, which has been infrequently detected in biological samples and has been found only in a few selected studies in urine and feces [22,46]. Lastly, hydroxybenzoic acids, including the important syringic acid, with various therapeutic uses [97], have been found across all types of samples (urine, plasma, feces) of participants that have consumed wine, while vanillic acid, 3,5-dihydroxybenzoic acid and 3-(3-hydroxyphenyl) propionic acid have been detected in both urine and feces. As an overall remark it is important to note that, although phenolic metabolites have been in the spotlight and have been thoroughly investigated in wine metabolomic studies, the results of the most recent non-targeted analysis studies indicate that other classes of metabolites, such as the endogenous metabolites, might be even more affected by wine consumption [24,25,34,44,60]; however, so far, these metabolites haven’t received the same level of attention.

### 4.2. Lipids and Lipid-like Molecules

A total of 84 lipid metabolites, related to wine consumption, have been identified in biological samples, mainly in plasma (Table 4). These lipid metabolites encompass a diverse array of categories, reflecting their wide-ranging chemical and biological functions. They were systematically categorized into thirteen (13) classes: short-chain fatty acids (SCFA), methyl-branched fatty acids, hydroxy fatty acids, medium-chain fatty acids (MCFA), long-chain fatty acids (LCFA), fatty acid esters, glycosylglycerols, fatty acyl glycosides, furanoid fatty acids, glycerophospholipids, glycerolipids, steroids and steroid derivatives, and monoterpenoids.

The majority of these lipids exhibited up-regulation, with a notable increase in certain microbial-derived metabolites, particularly short-chain fatty acids (SCFAs), as observed in the study by Belda in 2021 and Jiménez-Girón in 2015, 2014 [22,26,27]. Additionally, there were lipid metabolites, such as capric acid and arachidonic acid, which displayed correlations with wine consumption solely in an observational study, as reported by Pallister et al. in 2016 [63]. Moreover, 2,3-dihydroxyvaleric acid and sphingomyelins have been consistently detected in biological samples after wine intake, the first increasing and the second decreasing after wine-consumption as Haas et al. (2022) and Jacobs et al. (2012) reported [34,55]. 

While 2,3-dihydroxyvaleric acid’s origin remains relatively unexplored, sphingomyelins, in contrast, have undergone extensive investigation and are recognized for their pivotal role in regulating lifespan [98]. Additionally, it is worth noting that cholesterol and sphingomyelins’ levels exhibit a coordinated regulatory relationship [99].

### 4.3. Organic Acids and Derivatives

The organic acids and derivatives class includes a total of 40 reported compounds, with 24 identified as aliphatic organic acids and 15 as benzene organic acids and substituted derivatives (Table 5). Intriguingly, the majority of these metabolites exhibited an increase in concentration following wine consumption, suggesting a potential metabolic response triggered by wine intake. In contrast, only a limited subset of five metabolites showed a decrease after wine consumption, including formic acid, 2R,3R-dihydroxybutyric acid, dimethylguanidino valeric acid (DMGV), tricarballylic acid, and 3-indoxylsulfuric acid [27,34,50,60]. 

Among organic acid metabolites, 3-hydroxyphenylacetic acid, tartaric acid, 4-hydroxyhippuric acid, 4-hydroxyphenylacetic acid, and hippuric acid were consistently detected in multiple studies within the metabolome of participants after wine or wine derivative consumption. Tartaric acid is one of the principals acids found in wine [100], and a confirmed biomarker for wine consumption [41]. Equally noteworthy are the hydroxy-phenylacetic group compounds, with 3-hydroxyphenylacetic acid being a metabolite of rutin [101] and 4-hydroxyphenylacetic acid a metabolite of polymeric proanthocyanidins [102], alongside 3,4-dihydroxyphenylacetic acid, deriving from microbiota-driven quercetin metabolism [103]. Additionally, the hippuric group stands out, led by hippuric acid, a common urinary component that notably increases with the consumption of polyphenols, particularly (epi)catechin as present in tea [104,105]. This elevation is attributed to the transformation of these phenols into benzoic acid and subsequently into hippuric acid, excreted in urine. Moreover, 4-hydroxyhippuric acid serves as a secondary metabolite of hesperidin [106] and has been detected post-milk consumption with cocoa powder [107], while its isomer, 3-hydroxyhippuric acid, arises from flavonoids and hydroxycinnamates metabolism [108,109].

Overall, although the number of organic acid metabolites that have been identified in wine metabolomic studies is relatively limited, their presence in the human metabolome following wine intake is documented a total of 90 times across 51 metabolomic investigations, establishing this category as one of the most frequently detected groups of metabolites in the context of wine consumption.

### 4.4. Amino Acids and Derivatives

Across the 51 metabolomic studies, 38 amino acids and derivatives were reported to be influenced by wine consumption (Table 6). Notably, this group exhibited the highest proportion of down-regulated compounds, with nearly 50% of amino acids and their derivatives displaying down-regulation. Jacobs et al. (2012) reported reduced levels of tyrosine, threonine, and lysine in fasted plasma, consistent with a prior study indicating that polyphenols can influence protein digestibility [55]. Of particular interest, threonine was the only amino acid reported in two separate studies. Specifically, Jacobs et al. in 2012 observed a decrease in threonine levels after short-term (4 days) of wine derivative intake, while in Vázquez-Fresno et al. in 2016 noted a statistical difference before and after 28 days of wine consumption using ANOVA analysis [44,55]. The remaining 37 compounds were each reported only once, and their fluctuations related to wine intake.

### 4.5. Carbohydrates and Carbohydrate Conjugates

In the investigation of all biological samples, specifically urine and plasma, only 14 carbohydrate compounds were identified to exhibit statistical significance following wine consumption (Table 7). Most of them showed an increase in their content while only N-acetylneuraminate, which is involved in aminosugar metabolism according to Haas et al. (2022), was decreased [34].

Within this category, a mere two compounds stood out: mannitol [44,45,47] and scyllo-inositol [60,62,63], consistently detected across three distinct research papers. Mannitol is a polyol that is naturally found in wine and is formed by heterofermentative lactic acid bacteria through fructose reduction. According to Vazquez-Fresno in 2012, the presence of this polyol in urine samples following red wine drinking could be due to its fast removal from the body before undergoing metabolism [47]. Furthermore, scyllo-inositol has been linked to a variation in the SLC5A11 gene (rs4787294), which encodes a sodium-dependent glucose transporter that transports myo- and scyllo-inositol. SLC5A11 gene markers have been linked to susceptibility to systemic lupus erythematosus (SLE) [63]. 

### 4.6. Vitamins and Energy Compounds

Vitamins and energy-related compounds represent a smaller subset of metabolites that have been identified in recent metabolomic studies as being influenced by wine intake (Table 8). In these studies, only five compounds within this category were characterized, with analyses confined to plasma and urine samples [25,34,55]. Notable findings included an increase in nicotinic acid (a microbial fermentation product of aromatic amino acids) as well as an increase in pantoic acid (a precursor of vitamin B5) and isocitric lactone. [34] Additionally, a decrease in phosphate and cytidine triphosphate was reported [25]. 

### 4.7. Other Organic Compounds

Lastly, the assortment of 45 compounds that did not find placement in the preceding categories were placed in the “other organic compounds” class. Among these compounds, 12 were classified as aliphatic compounds, and the remaining 33 were identified as cyclic. These 33 cyclic compounds were further segmented into various subgroups, including organoheterocyclic, benzene and supstituted derivatives, pyrimidines, purines, and alkaloids. 

The fluctuation of these compounds in human metabolome after the consumption of wine is presented in Table 9. An up-regulation was noted in most of these organic compounds following wine intake. However, there were exceptions, such as the endogenous metabolite xanthine [26,27], and metabolites of different origins, like ajoene and cinnamyl cinnamate [25], which were reported to decrease. A particularly noteworthy finding was the down-regulation of stercobilin and urobilinogen, byproducts of bilirubin degradation, as elucidated by Jimenez in 2015 [27]. Urobilinogen remaining in the intestine is oxidized to form brown stercobilin, which imparts the characteristic color to feces. This finding aligns with numerous studies suggesting that flavan-3-ol-rich sources like wine may influence the intestinal microbiota by enhancing beneficial bacteria while inhibiting other groups, such as *Clostridium* spp. [26,27]. 

Finally, one of the most frequently encountered metabolites from this class, appearing in five distinct papers, was the microbial metabolite, 4-hydroxy-5-(phenyl)-valeric acid identified to be up-regulated in feces samples after moderate wine consumption [22,26,27,28,35]. Notably, this metabolite was also detected in feces samples post-cocoa consumption [110]. Moreover, as expected, ethanol and ethanol derivatives were detected [45,47]. 

## 5. Conclusions

The extensive review of 51 metabolomic studies led to some very interesting observations and conclusions. Noticeable alterations in the human metabolome were identified before and after wine consumption, following the metabolomic analysis of different biological samples and using various analytical techniques. This meticulous analysis of published metabolomic studies resulted to a comprehensive documentation and categorization of all metabolites that have been reported to appear in the human body after wine consumption or to be affected (increased or decreased) by it. This extensive list could be a valuable tool for researchers working in the field of wine metabolomics and could even constitute the keystone for the preparation of ready-to use MS databases, significantly facilitating non-target/suspect screening applications.

Furthermore, the distribution of metabolite categories in various bodily matrices reveals important insights. In general, urine stands out as the sample type with the highest number of detected metabolites and emerges as the primary matrix for identifying phenolic metabolites associated with wine consumption. When investigating lipids and amino acids and their derivatives, plasma is the matrix of choice, offering a suitable environment for lipid-related inquiries. Additionally, the presence of organic acids and their derivatives in urine, plasma, and feces samples suggests a dynamic equilibrium between metabolic pathways and excretion routes that can be modulated by wine consumption. In contrast, carbohydrates exhibit limited detection in these studies, likely due to their inherent resistance to the influence of wine consumption. This phenomenon arises due to the typically abundant presence of carbohydrates in the urine metabolome [111].

An intriguing observation also arises from studies conducted on individuals with good health versus those with underlying health conditions. In such a comparison, two compounds surfaced as being exclusive to studies involving individuals with health issues subsequent to wine consumption: mannitol and 3-methyl-2-oxovalerate showed a significant increase in urine and plasma samples after wine consumption from individuals with CAD, T2D or with ≥3 CHD risk factors [34,45,47]. Regarding the duration of the wine intake, no clear differentiation in the human metabolome is perceived after short-term and long-term wine consumption. The studies by Motilva et al. (2016), investigating into acute dealcoholized wine consumption [51], and Urpi-Sarda et al. (2015) on 4-week dealcoholized wine consumption [54], indicate that the concentrations of phenols in both blood and urine exhibit no statistically significant differences between long-term and short-term consumption periods. 

Overall, the findings presented in this review shed light on the intricate network of interconnected metabolites and pathways influenced by wine consumption, emphasizing the need for further research to comprehend their mechanistic relationships and overall health implications. It is imperative to underscore that the exploration of the interplay between wine and human health remains an ongoing endeavor, and this paper is expected to catalyze future investigations in the fields of metabolomics, biochemistry, and medicine within this domain.

## Figures and Tables

**Table 1:** Interventional Clinical Studies in wine metabolomics research.

Study Type	Intervention Period	Participants (Gender)	Health of Participants	Control Group	Wine (mL)/day	Type of Wine (Variety of Wine)	Matrix	Metabolomic Analysis	Analytical Technique (Mode)	Sample Preparation	Statistical Analysis	Ref.
crossover	20 days	8 (m, f)	healthy		272, 100 (gin)	RW, DRW, gin (Merlot)	feces	Targeted	UPLC-ESI-MS/MS (−)	dilution with NaCl and again with ACN	Student’s *t*-test, nonparametric Wilcoxon matched-pairs test, PCA	[35]
crossover	4 weeks	53 (m, f)	mildly hypertensive			capsules with mix of RW and GJ EXT	feces	Targeted	H-NMR	alkalized with NaOH and acidified with formic acid, LLE with D_2_O, CD_3_OD both containing TSP	Student’s *t*-test, PCA, PLS-DA	[36]
crossover	1 day	11 (m)	healthy		250	RW (Merlot)	plasma	Targeted	LC-ESI-MS/MS (±)	3 different methods tested [HLB SPE (selected), PPT with acidified MeOH, LLE with ethyl acetate]	-	[37]
crossover	1 day	12 (m)	healthy			RW, DRW, Phenol-stripped RW, water (Cabernet Shiraz)	plasma	Targeted	GC-MS	lyophilization, acetate buffer (pH 4.5), hydrolysis with b-glucuronidase, sulfatase, b-glucosidase, LLE with ethyl acetate, derivatization with BSTFA	ANOVA, general linear modelling	[38]
crossover	1 day	9 (m, f)	healthy		120	water, DRW, water and alcohol (ARW) (Cabernet sauvignon)	plasma	Targeted	GC-MS	hydrolysis with b-glucuronidase, arylsulfatase, LLE with methylene chloride/water and ethyl acetate, derivatization with BSTFA	Student’s *t* test, Wilcoxon signed-rank test, Fisher’s exact test	[39]
crossover	1 day	9 (m, f)	nm		120	RW, DRW (Cabernet Sauvignon)	plasma	Targeted	GC-MS	hydrolysis with β-glucuronidase, sulfatase, LLE with methylene chloride/water and ethyl acetate, derivatization with BSTFA	Least-squares nonlinear regression, ANOVA, Student’s *t*-test	[33]
crossover	3 weeks	42 (m)	CAD	-	250	RW (Merlot)	plasma	Non targeted	LC-MS/MS, UPLC-ESI-MS/MS (±)	PPT with MeOH	Shapiro-Wilk test, Student’s *t*-test, paired Wilcoxon test, Spearman rank correlation test, Bonferroni test	[34]
crossover	4 weeks	10 (m, f)	healthy		270	RW (nm)	plasma	Targeted	UHPLC-MS/MS (±)	3 different methods tested [PPT with ACN/formic acid/ammonium formate, HLB SPE, hybrid PPT]	Student’s *t*-test	[40]
crossover	1 day	21 (m)	healthy		100, 200, 300	RW (Tempranillo (85%), Graciano and Garnacha Tinta (15%))	urine	Targeted	LC–ESI-MS/MS (−)	dilution with formic acid/water	omnibus K2 D’Agostino-Pearson test, Shapiro-Wilk test, non-parametric Mann-Whitney U test, Wilcoxon test, Clopper-Pearson exact binomial method.	[41]
crossover	1 day	5 (m)	healthy		200	RW (Tempranillo)	urine	Targeted	LC−ESI-MS/MS (−)	dilution with water/formic acid	Student’s *t*-test	[42]
crossover	1 day	9 (m, f)	healthy		120	RW, DRW (Cabernet Sauvignon)	urine	Targeted	GC-MS	hydrolysis with β-d-glucuronidase, sulfatase, LLE with ethyl acetate, derivatization with BSTFA	Student’s *t*-test	[32]
crossover	1 day	6 (m)	healthy		500	RW, DRW, GJ (Lemberger)	urine	Targeted	HPLC-UV-Vis (+)	C18 SPE	ANOVA, Fischer’s test, linear regression analysis	[43]
crossover	4 weeks	57 (m, f)	T2D or ≥3 CHD risk factors			DRW (Merlot)	urine	Non targeted	H-NMR	Mixed with TSP, NaN_3_, KH_2_PO_4_ in D_2_O-buffer KOD (pH = 7)	OSC-PLS-DA	[44]
crossover	4 weeks	56 (m, f)	T2D or ≥3 CHD risk factors		272, 100 (gin)	RW, DRW, gin (nm)	urine	Non targeted	H-NMR	Mixed with TSP, NaN_3_, KH_2_PO_4_ in D_2_O	Mann-Whitney U test, Mann-Whitney test, logistic regression model	[45]
crossover	4 weeks	36 (m)	healthy		272	DRW (Merlot)	urine	Targeted	UPLC−MS/MS (−)	MCX SPE	Student’s *t*-test	[46]
crossover	4 weeks	61 (m)	T2D or ≥3 CHD risk factors		272, 100 (gin)	RW, DRW, gin (Merlot)	urine	Non targeted	H-NMR	Mixed with TSP, NaN_3_, KH_2_PO_4_ in D_2_O-buffer KOD (pH = 7)	ANOVA test, Fisher’s LSD test	[47]
crossover	4 weeks	10 (m)	T2D or ≥3 CHD risk factors		272	RW (Merlot), DRW	urine	Targeted	UPLC–MS/MS (−)	HLB SPE	Kolmogorov test, Levene test, nonparametric Friedman test, paired Wilcoxon test	[48]
crossover	4 weeks	58 (m, f)	mildly hypertensive			capsules 2:1 polyphenol-rich mix of RW and GJ EXTs, capsules with GJ EXT	urine	**GC-TOF-MS**: target, H-NMR: Non targeted	H-NMR, GC-TOF-MS	**H-NRM**: phosphate buffer (pH 7, TSP) **GC-MS**: hydrolysis with β-glucuronidase, LLE with ethyl acetate, derivatization with BSTFA/trimethylchlorosilane	Wilcoxon test, ML-PLS-DA	[49]
crossover	4 weeks	29 (m, f)	mildly hypertensive			capsules with mix of RW EXT and GJ EXT	urine	Non targeted	H-NMR	buffer phosphate and sodium salt (TSP)) (pH 3)	PLS-DA	[50]
crossover	1 day	12 (m, f)	healthy		272, 100 (gin)	DRW with EXT, DRW with encapsulated EXT, gin	urine, plasma	Targeted	UPLC-ESI-MS/MS (±)	HLB SPE	ANOVA, Tukey’s test	[51]
crossover	1 day	11 (m)	healthy		250, 1000 (GJ), 10 tablets	RW, GJ, capsules with RW EXT (nm)	urine, plasma	Targeted	GC-MS	**urine**: dilution with water, **plasma/urine**: acetate buffer (pH 5.2), hydrolysis with β-glucuronidase, LLE with ACN/ethyl acetate (in urine a solution of NaCl were added before extraction), derivatisedwith MSTFA: NH_4_I: 2-mercaptoethanol reaction mixture (ammonium iodide and 2-mercaptoethanol per litter of MSTFA)	least-square regression analysis	[52]
crossover	1 day	9 (m, f)	healthy		400	RW, GJ (Lemberger)	urine, plasma	Targeted	HPLC-UV-Vis (+)	C18 SPE	Shapiro-Wilk test, Student’s *t*-test	[53]
crossover	4 weeks	36 (m)	T2D or ≥3 of the CVRFs		272, 100 (gin)	RW, DRW (Merlot), gin	urine, plasma	Targeted	UPLC-ESI-MS/MS (−)	HLB SPE	PCA, HCA, ANOVA, Bonferroni test, Binary stepwise logistic regression analysis	[54]
crossover	5 days	35 (m)	healthy		630	capsules 2:1 polyphenol-rich mix of RW and GJ EXTs	urine, plasma	**H-NMR**: target, **GC-MS**, **LC-MS/MS**: target and Non targeted	GC-MS, LC-MS/MS (±), H-NMR	**plasma**: PPT with ACN, LLE with water/ethanol/dichloromethane. **urine**: dilution **H-NMR urine**: see [49] **GC-MS**: fatty acid esterification, derivatization with O-methylhydroxyamine hydrochloride, MSTFA	Mann-Whitney U test, Wilcoxon test	[55]
crossover	4 weeks	26 (m, f)	nm			capsules with mix of RW EXT and GJ EXT	urine, plasma, feces	Non targeted	GC–TOF–MS	**urine, plasma**: hydrolysis with β-d-glucuronidase, acidification with HCl, LLE with ethyl acetate, derivatization: BSTFA/TMCS	PCA, OPLS-DA	[56]
crossover	1 day	10 (nm)	nm			WW, GJ, vegetable cocktail (Lindemans Chardonnay)	urine, serum	Targeted	GC–MS	LLE with ethyl acetate, derivatization with BSTFA	Pearson test	[57]
crossover	4 weeks	52 (m, f)	healthy		200 (RW, WW), 300 (sparkling wine), 100 (gin)	RW, WW, gin (nm)	urine, serum	Targeted	LC-MS/MS (−)	HLB SPE	Kolmogorov test, Levene test, Wilcoxon test, Student’s *t*-test, ANOVA	[58]
parallel	4 weeks	19 (m, f)	healthy	abstention (5)	250	RW (nm)	feces	Targeted	UPLC-ESI-MS/MS (−), SPME-GCMS	DVB/CAR/PDMS SPME	Shapiro–Wilk test, Levene test, One-way ANOVA, Tukey’s test, Student’s *t*-test	[22]
parallel	4 weeks	8 (m, f)	healthy	abstention (4)	250	RW (Pinot Noir)	feces	Targeted	UPLC-ESI-MS/MS (−), SPME-GCMS	**phenolic metabolites**: dilution with NaCl and again with ACN/water **short-chain fatty acids**: SPE	Student’s *t*-test	[23]
parallel	4 weeks	41 (m, f)	healthy	abstention (8)	250	RW (Pinot Noir)	feces	Non targeted	UPLC-TOF-MS (−)	dilution (x2) with NaCl, filtered with polyvinylidene difluoride (PVDF) membrane	Shapiro-Wilk test, Student’s *t*-test, Wilcoxon matched-pairs test, PCA	[27]
parallel	4 weeks	41 (m, f)	healthy	abstention (8)	250	RW (Pinot Noir)	feces	Targeted and Non targeted	UPLC-ESI-MS/MS (−), UPLC-TOF/MS (−)	dilution with NaCl and again with ACN	Shapiro-Wilk test, Student’s *t*-test, Mann-Whitney test, Wilcoxon matched-pairs test, PCA	[26]
parallel	4 weeks	41 (m, f)	healthy		250	RW (Pinot Noir)	feces	Targeted	UPLC-ESI-MS/MS (−)	dilution with NaCl and again with ACN	Student’s *t*-test, Mann-Whitney test, Wilcoxon matched-pairs test, one-way ANOVA, Shapiro-Wilk test	[28]
parallel	15 days	20 (m, f)	healthy	Abstention (10)	300	RW, WW (nm)	plasma	Τargeted	HPLC-ESA	hydrolysis with glucuronidase/sulfatase, acetate buffer (pH 5.0), LLE with ethyl acetate	one-way ANOVA, non-parametric tests (Wilcoxon test, Kolmogorov-Smirnov test), Levene test, MANOVA, Bonferroni test, correlation analysis using Pearson’s test	[29]
parallel	1 day	25 (10, 5, 5 all m)	healthy	-	300, 600, 600	RW (Lambrusco, Cabernet Franc, Agliatico)	serum	Targeted	HPLC-UV-Vis (+), HPLC-MS (−), HPLC-MS/MS (−)	LLE with ethyl acetate	-	[30]
parallel	4 weeks	41 (m, f)	healthy	abstention (8)	250	RW (Pinot Noir)	urine	Non targeted	UPLC-QTOF-MS (−)	centrifugation and direct analysis	PCA, HCA, one-way ANOVA, OPLS-DA	[24]
parallel	4 weeks	41 (m, f)	healthy	abstention (8)	250	RW (Pinot Noir)	urine	Non targeted	UPLC-TOF-MS (−)	centrifugation and direct analysis	PCA, Student’s *t*-test, OPLS-DA	[25]
parallel	1 day	10 (m)	healthy	grape EXT tablets (3)	375	RW (Merlot)	urine, plasma	Targeted	LC–ESI–MS/MS (−)	HLB SPE	Mann–Whitney U test, Wilcoxon test	[31]

m: male, f: female, T2D: type 2 diabetes, CAD: coronary artery disease, CHD: coronary heart disease, CVRFs: cardiovascular risk factors, RW: regular red wine, WW: white wine, DRW: dealcoholized red wine, GJ: grape juice, EXT: extract, PPT: protein precipitation, DVB/CAR/PDMS: Divinylbenzene/Carboxen/Polydimethylsiloxane, ACN: acetonitrile, MeOH: methanol, BSTFA: N,O-bis (trimethylsilyl)trifluoroacetamide, TSP: 3-(trimethylsilyl)-proprionate-2,2,3,3-d_4_, NaN_3_: sodium azide, D_2_O: deuterium water, KOD: potassium deuteroxide, CD_3_OD: deuterated methanol, LC: liquid chromatography, MS/MS: tandem mass spectrometry, UPLC: ultra-high performance liquid chromatography, SPME: solid phase microextraction, DDA: data dependent acquisition, MRM: multiple reaction monitoring, PCA: Principal component analysis, ANOVA: Analysis of Variance, MANOVA: Multivariate analysis of variance, PLS-DA: Partial Least Squares Discriminant Analysis OPLS-DA: Orthogonal Partial Least Squares Discriminant Analysis, OSC-PLS-DA: Orthogonal Signal Correction-Partial Least Squares Discriminant Analysis, HCA: Hierarchical Clustering Analysis, PC-PR2: principal component partial R-square analysis, GLMs: general linear models, FDR: false discovery rate, LASSO: least absolute shrinkage and selection operator, LSD: Least Significant Difference, nm: not mentioned.

**Table 2:** Observational Clinical Studies in wine metabolomics research.

Number of Participants (Gender)	Health of Participants	Matrix	Metabolomic Analysis	Analytical Technique (Mode)	Sample Preparation	Statistical Analysis	Ref.
671 (m, f)	general population	plasma	Targeted	UPLC-ESI-MS/MS (±)	PPT with MeOH	Pearson’s partial correlation	[59]
1157 (m, f)	T2D or ≥3 major CVRFs	plasma	Non targeted	UPLC-MS/MS (−)	**amino acids and other polar metabolites**: LLE with ACN/MeOH/formic acid, **lipids**: LLE with isopropanol	cross-validation	[60]
25 (nm)	healthy	plasma	Targeted	HPLC-ESI-MS/MS (± tested but–preferred)	PPT with acetic acid, HLB SPE	not used a statistical analysis test rather than statistical parameters	[61]
502 (m, f)	healthy	plasma	Non targeted	UPLC-ESI-MS/MS (±)	PPT with MeOH	2-sided statistical tests (chi-square test, Mann-Whitney U Test), FDRs, LASSO regression	[62]
3559 (f)	general population	plasma, serum	Targeted and Non targeted	UPLC-MS/MS (±), GC-MS	**UPLC**: PPT with MeOH, **GC**: PPT with MeOH, derivatization: BSTF	linear regression analysis	[63]
849 (m, f)	general population	serum	Non targeted	UPLC-ESI-MS/MS (−)	LLE with MeOH	Student’s *t*-tests, chi-square test	[64]
222 (m, f)	T2D or ≥3 major CVRFs	urine	Targeted	LC–ESI-MS/MS (−)	dilution with water/formic acid	Shapiro-Wilk test, multiple adjusted linear regression models	[65]
475 (m, f)	general population	urine	Targeted	UHPLC-ESI-MS/MS (+)	hydrolysis with β-glucuronidase, sulfatase, LLE with ethyl acetate	one-way ANOVA, Spearman’s rank correlations, partial Spearman’s correlations	[66]
475 (m, f)	general population	urine	Targeted	UPLC-ESI-MS-MS (+)	hydrolysis with β-glucuronidase, sulfatase LLE with ethyl acetate	expectation-maximization (EM) algorithm, GLMs, RRR-VIP method, reduced rank regression, LASSO regression, RRR analysis, internal two-fold cross-validation	[67]
1386 (m, f)	general population	urine	Targeted	UPLC-ESI-MS/MS	hydrolysis with β-glucuronidase, sulfatase LLE with ethyl acetate	PC-PR2, one-way ANOVA	[68]
253 (m, f)	new or recurrent colorectal adenoma cases and adenoma-free controls	urine	Non targeted	UPLC-MS, UPLC-ESI-MS/MS (±), GC-MS	direct analysis	Wilcoxon’s signed rank test, chi-square test, Partial Pearson correlation, FDR calculation (Benjamini-Hochberg procedure), LASSO regression	[69]
481 (m, f)	general population	urine	Non targeted	UPLC-QTOF-MS (−)	dilution with water	OPLS-DA, HCA	[70]
1000 (m, f)	T2D or ≥3 CHD risk factors	urine	Targeted	UPLC-MS/MS (−)	HLB SPE	chi-square tests, Kolmogorov test, Levene test, one way ANOVA, Mann-Whitney test	[71]
1369 (f)	healthy postmenopausal women	serum, urine	Non targeted	UPLC-ESI-MS/MS (±)	PPT with MeOH	Pearson’s partial correlation, linear Support Vector Machine multivariate classification model	[72]

m: male, f: female, T2D: type 2 diabetes, CAD: coronary artery disease, CHD: coronary heart disease, CVRFs: cardiovascular risk factors PPT: protein precipitation, DVB/CAR/PDMS: Divinylbenzene/Carboxen/Polydimethylsiloxane, ACN: acetonitrile, MeOH: methanol, BSTFA: N,O-bis (trimethylsilyl)trifluoroacetamide, TSP: 3-(trimethylsilyl)-proprionate-2,2,3,3-d_4_, NaN_3_: sodium azide, D_2_O: deuterium water, KOD: potassium deuteroxide, CD_3_OD: deuterated methanol, LC: liquid chromatography, MS/MS: tandem mass spectrometry, UPLC: ultra-high performance liquid chromatography, SPME: solid phase microextraction, DDA: data dependent acquisition, MRM: multiple reaction monitoring, PCA: Principal component analysis, ANOVA: Analysis of Variance, MANOVA: Multivariate analysis of variance, PLS-DA: Partial Least Squares Discriminant Analysis OPLS-DA: Orthogonal Partial Least Squares Discriminant Analysis, OSC-PLS-DA: Orthogonal Signal Correction-Partial Least Squares Discriminant Analysis, HCA: Hierarchical Clustering Analysis, PC-PR2: principal component partial R-square analysis, GLMs: general linear models, FDR: false discovery rate, LASSO: least absolute shrinkage and selection operator, LSD: Least Significant Difference, nm: not mentioned.

**Table 3:** Phenolic metabolites affected by wine consumption.

	Effect of Wine Consumption	Name	Molecular Formula	Sample	Analytical Technique	Ref.
	up	Phenol sulfate	C_6_H_6_O_4_S	urine	UPLC-QTOF-MS	[25]
		Quinic acid	C_7_H_12_O_6_	urine	UPLC-QTOF-MS	[24]
**Phenylpropanoic acids**		3-(4-hydroxyphenyl)lactate	C_9_H_9_O_4_-	plasma	UPLC-MS/MS, GC-MS	[63]
		3-hydroxyphenylpropionic acid	C_9_H_10_O_3_	urine	H-NMR, GC-TOF-MS	[49]
		Vanillactic acid	C_10_H_12_O_5_	plasma	LC-MS/MS, UPLC-MS/MS	[34]
	up	Homovanillic acid	C_9_H_10_O_4_	urine	UPLC-MS/MS, GC-MS, GC–TOF–MS	[34,49,56]
	down	Homovanillic acid sulfate	C_9_H_10_O_7_S	urine	UPLC-QTOF-MS	[25]
		4-hydroxymandelic acid	C_8_H_8_O_4_	urine	GC-MS, GC–TOF–MS	[49,56]
	down	4-methoxyphenylethanol sulfate	C_9_H_12_O_6_S	urine	UPLC-QTOF-MS	[25]
**Gallic acid metabolites**		**Gallic acid**	C_7_H_6_O_5_	urine, plasma	UPLC-ESI-MS-MS, UPLC−MS/MS	[46,54,76]
		Gallic acid sulfate	C_7_H_6_O_8_S	urine	UPLC-MS/MS	[51]
		Gallic acid glucuronide	C_13_H_16_O_10_	urine	UPLC-MS/MS	[51]
	up	**4-O-methylgallic acid**	C_8_H_8_O_5_	urine, plasma	UPLC-QTOF-MS, UPLC−MS/MS, GC-MS	[38,46,70]
	up	**3-O-methylgallic acid**	C_8_H_8_O_5_	feces	UPLC-ESI-MS/MS, UPLC-TOF-MS	[26,28,35]
		**Methylgallic acid sulfate**	C_8_H_8_O_8_S	urine	UPLC-QTOF-MS, UPLC−MS/MS	[46,54,70]
	up	Gallic acid ethyl ester	C_9_H_10_O_5_	urine	UPLC-ESI-MS/MS	[67,68]
		Gallic acid ethyl ester sulfate	C_9_H_10_O_8_S	urine	UPLC-QTOF-MS	[70]
		Ethylgallate	C_9_H_10_O_5_	urine	UPLC−MS/MS	[46,54]
		Ethylgallate glucuronide 1,2	C_15_H_18_O_11_	urine	UPLC−MS/MS	[46]
		Ethylgallate sulfate	C_9_H_10_O_8_S	urine	UPLC−MS/MS	[46,54]
		Pyrogallol (1,2,3-trihydroxybenzene)	C_6_H_6_O_3_	urine	UPLC−MS/MS, GC-MS, GC-MS, LC-MS/MS	[46,49,55]
	up	Pyrogallol sulfate	C_6_H_6_O_6_S	urine	UPLC-QTOF-MS	[25]
	up	Phloroglucinol (1,3,5-trihydroxybenzene)	C_6_H_6_O_3_	urine	UPLC-QTOF-MS	[25]
**Tyrosols**		Hydroxytyrosol	C_8_H_10_O_3_	urine	UPLC-ESI-MS-MS, UPLC-TOF-MS	[25,51,68]
		Hydroxytyrosol sulfate	C_8_H_10_O_6_S	urine	UPLC-QTOF-MS, UPLC-ESI-MS/MS	[51]
	down	Hydroxytyrosol glucoside	C_14_H_20_O_8_	urine	UPLC-QTOF-MS	[25]
		Tyrosol	C_8_H_10_O_2_	urine	UPLC-ESI-MS/MS	[68]
	up	Tyrosol sulfate	C_8_H_10_O_5_S	urine	UPLC-TOF-MS	[25]
**Hydroxyhippuric acids**		Vanilloylglycine	C_10_H_11_NO_5_	urine	UPLC-MS/MS	[46]
**Cinnamic acids and derivatives**		(Z)-N-Feruloyl-5-hydroxyanthranilic acid	C_17_H_15_NO_6_	urine	UPLC-QTOF-MS	[24]
**Hydrocinnamic acids**	up	Coumaroyl-glucose	C_15_H_18_O_8_	urine	UPLC-QTOF-MS	[25]
		Ferulic acid/Isoferulic acid	C_10_H_10_O_4_	urine	UPLC−MS/MS, LC-MS/MS, GC-MS	[46,49,55]
	up	Ferulic/isoferulic acid sulfate	C_10_H_10_O_7_S	urine	UPLC-ESI-MS/MS, UPLC-TOF-MS	[25,51]
		Ferulic acid glucuronide	C_16_H_18_O_10_	urine	UPLC-MS/MS	[51]
		Dihydroferulic acid	C_10_H_12_O_4_	urine	H-NMR, GC-TOF-MS	[49]
	up	**Caffeic acid**	C_9_H_8_O_4_	urine, plasma	UPLC−MS/MS, UPLC-TOF-MS, HPLC-ESA	[25,29,38,46]
	down	Caffeic acid sulfate	C_9_H_8_O_7_S	urine	UPLC−MS/MS, UPLC-TOF-MS, HPLC-ESA	[25,51]
		Dihydrocaffeic acid	C_9_H_10_O_3_	urine	UPLC−MS/MS	[46]
	down	Dihydrocaffeic acid 3-sulfate	C_9_H_10_O_7_S	urine	UPLC-QTOF-MS	[25]
		Sinapic acid	C_11_H_12_O_5_	urine	UPLC−MS/MS	[46]
		**p-coumaric**	C_9_H_8_O_3_	urine, plasma, feces	UPLC-MS/MS, UPLC-ESI-MS/MS	[35,46,54,68]
		**m-coumaric**	C_9_H_8_O_3_	urine, feces	UPLC-ESI-MS/MS	[22,46]
		m-coumaric acid sulfate	C_9_H_8_O_6_S	urine	UPLC-QTOF-MS	[70]
	down	Piplartine	C_17_H_19_NO_5_	urine	UPLC-QTOF-MS	[25]
**Hydroxybenzoic acid derivatives**		3-hydroxybenzoic acid	C_7_H_6_O_3_	urine	UPLC−MS/MS	[46]
		4-hydroxybenzoic acid	C_7_H_6_O_3_	urine, plasma	UPLC−MS/MS, GC-MS	[46,49]
		2,4-dihydroxybenzoic acid	C_7_H_6_O_4_	urine	UPLC−MS/MS	[46,54]
	up	2,5-dihydroxybenzoic acid (gentisate)	C_7_H_6_O_4_	urine, plasma	UPLC−MS/MS	[34,46]
		2,6-dihydroxybenzoic acid	C_7_H_6_O_4_	urine	UPLC−MS/MS	[46]
	up	**3,5-dihydroxybenzoic acid**	C_7_H_6_O_4_	urine, feces	UPLC-ESI-MS/MS, UPLC-TOF-MS	[26,28,35,46]
		**3-(3-hydroxyphenyl)propionic acid or 3-(4-hydroxyphenyl)propionic acid**	C_9_H_10_O_3_	urine, feces	UPLC-QTOF/MS, UPLC-ESI-MS/MS, GC–TOF–MS	[22,26,27,46,56]
		3-(3-hydroxyphenyl)-3-hydroxypropionic acid	C_9_H_10_O_4_	urine	GC-MS, GC–TOF–MS	[49,56]
	up	**Vanillic acid** (3-methoxy-4-hydroxybenzoic acid)	C_8_H_8_O_4_	urine, feces	UPLC−MS/MS, GC-MS, GC–TOF–MS, UPLC-ESI-MS/MS	[26,28,35,46,49,55,56]
		Isovanillic acid (4-methoxy-3-hydroxybenzoic acid)	C_8_H_8_O_4_	urine	H-NMR, GC-TOF-MS	[49]
	up	Vanillic acid 4-sulfate	C_8_H_8_O_7_S	urine	UPLC-QTOF-MS	[25]
	up	Protocatechuic acid	C_7_H_6_O_4_	urine, plasma, feces	UPLC-MS/MS, UPLC-ESI-MS/MS, UPLC-TOF-MS	[26,28,35]
		Protocatechuic acid sulfate	C_7_H_6_O_7_S	urine	UPLC-MS/MS	[51]
		**Syringic acid** (4-hydroxy-3,5-dimethoxybenzoic acid)	C_9_H_10_O_5_	urine, plasma, feces	UPLC−MS/MS, GC-MS, GC–TOF–MS, UPLC-ESI-MS/MS, UPLC-TOF-MS	[26,28,35,46,49,56]
		Syringic acid sulfate	C_9_H_10_O_8_S	urine	UPLC-ESI-MS/MS, UPLC-QTOF-MS	[51,70]
		Syringic acid glucuronide	C_15_H_18_O_11_	urine	UPLC-MS/MS	[51]
	down	Methyl 3-(2,3-dihydroxy-3-methylbutyl)-4-hydroxybenzoate (Hostmaniane)	C_13_H_18_O_5_	urine	UPLC-QTOF-MS	[25]
**Hydroxycoumarins**		4-hydroxycoumarin	C_9_H_6_O_3_	plasma	LC-MS/MS, UPLC-MS/MS	[34]
**Coumarins and derivatives**		Urolithin A	C_13_H_8_O_4_	urine	UPLC-QTOF-MS	[24]
		Cis-caffeoyl tartaric acid/caftaric acid	C_13_H_12_O_9_	urine	UPLC-QTOF-MS	[24]
	up	Aurantricholide B	C_17_H_10_O_6_	urine	UPLC-QTOF-MS	[24]
		5-(6-hydroxy-3,7-dimethyl-2,7-octadienyloxy)-7-methoxycoumarin	C_20_H_24_O_5_	urine	UPLC-QTOF-MS	[24]
**Catechols**		Catechol/pyrocatechol	C_6_H_6_O_2_	urine, feces	LC-MS/MS, UPLC-ESI-MS/MS, GC-MS	[22,55]
	up	(Pyro) catechol sulfate	C_6_H_6_O_5_S	urine	UPLC-QTOF-MS	[25]
		3-methoxycatechol sulfate	C_7_H_6_O_7_S	plasma	LC-MS/MS, UPLC-MS/MS	[34]
	up	O-methoxycatechol-o-sulfate	C_7_H_8_O_5_S	urine	UPLC-QTOF-MS	[25]
		3,4-dihydrophenylglycol	C_8_H_10_O_4_	urine	GC-MS, LC-MS/MS, H-NMR	[55]
	up	4-hydroxy-5-(3-hydroxyphenyl)-valeric acid	C_11_H_14_O_4_	feces	UPLC-QTOF-MS, UPLC-ESI-MS/MS	[26,27]
	up	4-hydroxy-5-(3,4-dihydroxyphenyl)-valeric acid	C_11_H_12_O_5_	feces	UPLC-QTOF-MS, UPLC-ESI-MS/MS	[26,28]
	up	4-hydroxy-5-(3,4-dihydroxyphenyl)-valeric acid-o-sulfate	C_11_H_14_O_8_S	urine	UPLC-QTOF-MS	[25]
		4-hydroxy-5-(3,4-dihydroxyphenyl)-valeric acid-o-methyl-o-sulfate	C_12_H_16_O_9_S	urine	UPLC-QTOF-MS	[24]
	up	**5-(3,4-dihydroxyphenyl)-γ-valerolactone (DHPV 1,2)**	C_11_H_12_O_4_	urine, feces	UPLC-QTOF/MS, UPLC-ESI-MS/MS	[26,27,46,51]
	up	5-(3,4-dihydroxyphenyl)-γ-valerolactone glucuronides	C_17_H_20_O_10_	urine	UPLC−MS/MS, UPLC-TOF-MS	[25,46]
		5-(3,4-dihydroxyphenyl)-γ-valerolactone sulfates	C_11_H_12_O_7_S	urine	UPLC−MS/MS, UPLC-TOF-MS	[25,46]
		Methoxyhydroxyphenyl-γ-valerolactone (MHPV)	C_12_H_14_O_4_	urine	UPLC-MS/MS	[46]
		Methoxyhydroxyphenyl-γ-valerolactone glucuronide	C_18_H_22_O_10_	urine	UPLC-MS/MS	[46]
		Methoxyhydroxyphenyl-γ-valerolactone sulfates	C_12_H_14_O_7_S	urine	UPLC-MS/MS	[46]
	up	5-(3′-hydroxyphenyl)-γ-valerolactone or 5-(4′-hydroxyphenyl)-γ-valerolactone	C_12_H_12_O_2_	feces	UPLC−MS/MS, UPLC-TOF-MS	[26,28]
	up	5-(3′,4′,5′-trihydroxyphenyl)-γ-valerolactone-o-methyl-o-sulfate	C_12_H_14_O_9_S	urine	UPLC-QTOF-MS	[25]
**Stilbenes**		**Resveratrol**	C_14_H_12_O_3_	urine, plasma, serum	UPLC-ESI-MS/MS, GC-MS, HPLC-ESA, HPLC-UV-Vis, UPLC-MS/MS	[29,30,52,54,57,61,66,67,68]
		Resveratrol sulfate	C_14_H_12_O_6_S	urine, plasma	UPLC-ESI-MS/MS, UPLC-MS/MS	[40,48,51,71]
		Resveratrol 3,4′-disulfate	C_14_H_12_O_9_S_2_	urine	LC–ESI–MS/MS	[31]
		Resrveratrol glucoside (trans-resveratrol 3-O-b-glucoside, or cis-resveratrol 3-O-b-glucoside)	C_20_H_22_O_8_	urine	UPLC-MS/MS, LC–ESI–MS/MS, UPLC-ESI-MS/MS	[37,48,54,66]
		Resveratrol glucoside sulfate	C_20_H_22_O_11_S	urine	UPLC-MS/MS, LC–ESI–MS/MS	[48]
		Resveratrol glucuronide (trans-resveratrol 3-O-glucuronide or trans-resveratrol 4-O-glucuronide or cis-resveratrol 3-O-glucuronide or cis-resveratrol 4-O-glucuronide)	C_20_H_20_O_9_	urine, plasma	UPLC-ESI-MS/MS, LC-MS/MS, LC–ESI–MS/MS	[37,48,51,58,71]
		Dihydroresveratrol (DHR)	C_14_H_14_O_3_	urine, plasma, serum	LC–ESI–MS/MS, GC-MS	[48,52]
		Dihydroresveratrol glucuronide	C_20_H_22_O_9_	urine	UPLC-QTOF-MS, LC–ESI–MS/MS	[48,70]
		Dihydroresveratrol sulfate	C_14_H_14_O_6_S	urine	LC–ESI–MS/MS, UPLC-MS/MS	[40,48]
**Flavonoids**	up	**(Epi)catechin**	C_15_H_14_O_6_	urine, plasma, serum	UPLC-ESI-MS/MS, HPLC-ESA, GC–MS, UPLC-QTOF-MS	[24,29,32,33,57,68]
	up	(Epi)catechin glucuronides	C_21_H_22_O_12_	urine	UPLC−MS/MS, GC-MS	[32,46]
	up	(Epi)catechin sulfates	C_15_H_14_O_9_S	urine, plasma	UPLC−MS/MS, GC-MS	[32,33,46,51]
	up	Methyl catechin	C_16_H_16_O_6_	urine, plasma	GC-MS	[32,33]
	up	Methyl catechin glucuronide sulfates (1,2,3)		urine	GC-MS	[32]
	up	Methyl(epi)catechin glucuronides (1,2,3)	C_22_H_24_O_12_	urine	UPLC-ESI-MS/MS, UPLC-MS/MS, GC-MS	[32,46,51]
	up	Methyl(epi)catechin sulfates (1,2,3)	C_16_H_16_O_9_S	urine	UPLC-ESI-MS/MS, UPLC-MS/MS, GC-MS	[32,46,51]
	up	Catechin glucuronide sulfate		urine	GC-MS	[32]
		Quercetin	C_15_H_10_O_7_	urine, serum	GC–MS	[57]
	down	Quercetin o-(acetyl-glucoside)	C_23_H_22_O_13_	urine	UPLC-QTOF-MS	[25]
		Cyanidin 3-glucoside	C_21_H_21_ClO_11_	urine, plasma	HPLC-UV-Vis	[53]
		Delphinidin 3-glucoside	C_21_H_21_O_12_+	urine, plasma	HPLC-UV-Vis	[53]
		Peonidin 3-glucoside	C_22_H_23_ClO_11_	urine, plasma	HPLC-UV-Vis	[53]
		Petunidin 3-glucoside	C_22_H_23_O_12_+	urine, plasma	HPLC-UV-Vis	[53]
		Malvidin glucoside	C_23_H_25_ClO_12_	urine	UPLC-ESI-MS/MS, LC-UV-VIS	[51,53]
		Procyanidin b-type dimer	C_30_H_26_O_12_	urine	UPLC-QTOF-MS	[24]
	down	Naringenin	C_15_H_12_O_5_	urine	UPLC-QTOF-MS	[25]
	up	Luteolin sulfate	C_15_H_10_O_9_S	urine	UPLC-QTOF-MS	[25]
	up	Hesperetin-o-sulfate	C_16_H_14_O_9_S	urine	UPLC-QTOF-MS	[25]
	up	5,7-dihydroxy-3′,4′-dimethoxy-5′-prenylflavanone	C_22_H_24_O_6_	urine	UPLC-QTOF-MS	[25]
	up	5′-methoxybilobetin	C_32_H_22_O_11_	urine	UPLC-QTOF-MS	[25]
	up	Hordatine B glucoside	C_35_H_50_N_8_O_10_	urine	UPLC-QTOF-MS	[25]
**Isoflavonoids**	up	Kanzonol I	C_27_H_32_O_5_	urine	UPLC-QTOF-MS	[25]
	up	Kanzonol R	C_22_H_26_O_5_	urine	UPLC-QTOF-MS	[25]
**Phenylpropanoids and polyketides**	up	Licarin C	C_22_H_26_O_5_	urine	UPLC-QTOF-MS	[25]
**Lignans, neolignans and related compounds**		Enterolactone	C_18_H_18_O_4_	urine	UPLC−MS/MS	[46]
	down	Tracheloside	C_27_H_34_O_12_	urine	UPLC-QTOF-MS	[25]
	up	Azaspirazid	C_47_H_71_NO_12_	urine	UPLC-QTOF-MS	[25]
**Others**	up	4′,6′-dihydroxy-2′-methoxyacetophenone 6′-glucoside	C_15_H_20_O_9_	urine	UPLC-QTOF-MS	[25]
		4-methylbenzenesulfonate	C_7_H_7_O_3_S-	plasma	LC-MS/MS, UPLC-MS/MS	[34]
	up	alpha-Terpinyl cinnamate	C_19_H_24_O_2_	urine	UPLC-QTOF-MS	[25]

**Table 4:** Lipids and lipid-like molecules affected by wine consumption.

	Effect of Wine Consumption	Name	Molecular Formula	Sample	Analytical Tecnhique	Ref.
**Short-chain fatty acid (SCFA)**	up	Butyric acid	C_4_H_8_O_2_	feces	UPLC-ESI-MS/MS, SPME-GC-MS	[22,23]
	up	Acetic acid	C_2_H_4_O_2_	feces	UPLC-ESI-MS/MS, SPME-GC-MS	[22,23]
	up	Propionic acid	C_3_H_6_O_2_	feces	UPLC-ESI-MS/MS, SPME-GC-MS	[22,23]
**Methyl-branched fatty acids**	up	2-methylbutyric acid	C_5_H_10_O_2_	feces	UPLC-QTOF-MS, UPLC-ESI-MS/MS	[26,27]
		Valeric acid	C_5_H_10_O_3_	feces	UPLC-ESI-MS/MS, SPME-GCMS	[22]
	up	Isovaleric acid	C_5_H_10_O_2_	feces	UPLC-ESI-MS/MS, SPME-GC-MS	[22,23]
**Hydroxy fatty acids**		Alpha-hydroxyisovalerate	C_5_H_10_O_3_	plasma	LC-MS, GC-MS, HPLC-MS/MS	[34,63]
		3-hydroxyisovaleric acid	C_5_H_10_O_3_	plasma	UPLC-MS/MS, GC-MS	[63]
	up	**2,3-dihydroxyvaleric acid**	C_5_H_10_O_4_	plasma, urine	UPLC-MS/MS, GC-MS, UPLC-QTOF-MS	[25,34,59,69,72]
	up	2,3-dihydroxy-3-methylvaleric acid	C_6_H_12_O_4_	urine	UPLC-QTOF-MS	[24,25]
	up	Citramalic acid	C_5_H_8_O_5_	plasma	LC-MS/MS, UPLC-MS/MS	[34]
	up	2-isopropylmalate	C7H10O4	urine	UPLC-MS/MS, GC-MS	[24,69]
		Isopropylmalic acid	C_7_H_12_O_5_	urine	UPLC-QTOF-MS	[24]
		3-hydroxymethylglutaric acid	C_6_H_10_O_5_	urine	UPLC-QTOF-MS	[24]
		2,3-dimethyl-3-hydroxyglutaric acid	C_7_H_12_O_5_	urine	UPLC-QTOF-MS	[24,25]
**Branched fatty acids**	up	Diethylmalonic acid	C_7_H_12_O_4_	feces	UPLC-QTOF-MS, UPLC-ESI-MS/MS	[26,27]
**Medium-chain fatty acids (MCFA)**		Caproic acid	C_6_H_12_O_2_	feces	UPLC-ESI-MS/MS, SPME-GCMS	[22]
**Long-chain fatty acids (LCFA)**		Hydroxyoctanoic acid	C_8_H_16_O_3_	plasma	LC-MS/MS, UPLC-MS/MS	[34]
		Octanoic acid (Caprylic acid)	C_8_H_16_O_2_	feces	UPLC-ESI-MS/MS, SPME-GCMS, UPLC-MS/MS, GC-MS	[22,75]
		Capric acid (10:0)	C_10_H_20_O_2_	plasma	UPLC-MS/MS, GC-MS	[63]
	down	3-hydroxyoctadecanoic acid	C_18_H_36_O_3_	plasma	LC-MS/MS, UPLC-MS/MS	[34]
		Decanoic acid	C_10_H_20_O_2_	feces	UPLC-ESI-MS/MS, SPME-GCMS	[22]
		Docosahexaenoic acid (DHA; 22:6n3)	C_22_H_32_O_2_	plasma	UPLC-MS/MS, GC-MS	[63]
		Arachidonic acid (20:4n6)	C_20_H_32_O_2_	plasma	UPLC-MS/MS, GC-MS	[63]
		Eicosapentaenoic acid (epa; 20:5n3)	C_20_H_30_O_2_	plasma	UPLC-MS/MS, GC-MS	[63]
		Oleic acid	C_18_H_34_O_2_	plasma	GC-MS, LC-MS/MS, H-NMR	[55]
		Docosapentaenoate (n3 DPA; 22:5n3)	C_22_H_34_O_2_	plasma	UPLC-MS/MS, GC-MS	[63]
		Hexadecenedioate (C16:1-DC)	C_16_H_28_O_4_	plasma	LC-MS/MS, UPLC-MS/MS	[34]
		Octadecenedioate (C18:1-DC)	C_18_H_32_O_4_	plasma	LC-MS/MS, UPLC-MS/MS	[34]
**Lineolic acids and derivatives**		9- and 13-hydroxyoctadecadienoic acids (HODES)	C_18_H_32_O_3_	plasma	LC-MS/MS, UPLC-MS/MS	[34]
		Stearidonate (18:4n3)	C_18_H_27_O_2_^−^	plasma	UPLC-MS/MS, GC-MS	[63]
**Fatty acid esters**	up	2-phenethyl butyrate	C_12_H_16_O_2_	feces	UPLC-QTOF-MS, UPLC-ESI-MS/MS	[26,27]
	up	2-phenylethyl hexanoate	C_14_H_20_O_2_	feces	UPLC-QTOF-MS, UPLC-ESI-MS/MS	[26,27]
	up	Docosahexaenoic acid methyl ester	C_23_H_34_O_2_	feces	UPLC-QTOF-MS, UPLC-ESI-MS/MS	[26,27]
		Dodecadienoate (12:2)	C_19_H_34_O_3_	plasma	LC-MS/MS, UPLC-MS/MS	[34]
**Fatty acids esters (Carnitines)**		Acetylcarnitine	C_9_H_17_NO_4_	plasma	LC-MS/MS, UPLC-MS/MS	[34]
	down	3-decenoylcarnitine	C_17_H_31_NO_4_	plasma	LC-MS/MS, UPLC-MS/MS	[34]
		3-hydroxyhexanoylcarnitine	C_13_H_25_NO_5_	plasma	LC-MS/MS, UPLC-MS/MS	[34]
		5-dodecenoylcarnitine (C12:1)	C_19_H_35_NO_4_	plasma	LC-MS/MS, UPLC-MS/MS	[34]
		Adipoylcarnitine	C_13_H_24_NO_6_	plasma	LC-MS/MS, UPLC-MS/MS	[34]
		Octadecanedioylcarnitine (C18-DC) or octadecenedioylcarnitine (C18:1-DC)	C_25_H_47_NO_4_	plasma	LC-MS/MS, UPLC-MS/MS	[34]
		Succinylcarnitine	C_11_H_19_NO_6_	plasma	LC-MS/MS, UPLC-MS/MS	[34]
		Lignoceroylcarnitine (C24)	C_31_H_61_NO_4_	plasma	LC-MS/MS, UPLC-MS/MS	[34]
		Cis-4-decenoylcarnitine (C10:1)	C_17_H_32_NO_4_	plasma	LC-MS/MS, UPLC-MS/MS	[34]
**Glycosylglycerols**	up	Galactosylglycerol	C_9_H_18_O_8_	urine	UPLC-QTOF-MS	[25]
**Fatty acyl glycosides**	up	(3s,5r,6s,7e,9x)-7-megastigmene-3,6,9-triol 9-glucoside	C_19_H_34_O_8_	urine	UPLC-QTOF-MS	[25]
	up	Methyl helianthenoate f glucoside	C_17_H_22_O_8_	urine	UPLC-QTOF-MS	[25]
	down	4-methoxybenzenepropanol 1-(2-sulfoglucoside)	C_16_H_24_O_10_S	urine	UPLC-QTOF-MS	[25]
**Furanoid fatty acids**	up	Wyeronic acid	C_13_H_10_O_4_	urine	UPLC-QTOF-MS	[25]
		3-carboxy-4-methyl-5-pentyl-2-furanpropanoic acid (3-CMPFP)	C_14_H_20_O_5_	urine	UPLC-QTOF-MS	[25]
		3-carboxy-4-methyl-5-propyl-2-furanpropanoate (CMPF)	C_12_H_16_O_5_	plasma	UPLC-MS/MS	[72]
**Glycerophospholipids**		Glycosyl-n-behenoyl-sphingadienine (d18:2/22:0)		plasma	LC-MS/MS, UPLC-MS/MS	[34]
	down	Linoleoyl-linolenoyl-glycerol (18:2/18:3), (18:2/18:2), (18:2/18:2)	C_39_H_66_O_5_	plasma	LC-MS/MS, UPLC-MS/MS	[34]
	down	Oleoyl-linoleoyl-glycerol	C_39_H_70_O_5_	plasma	UPLC-MS/MS	[72]
		1-(1-enyl-palmitoyl)-2-palmitoyl-GPC (p-16:0/16:0)	C_40_H_80_NO_7_P	plasma	LC-MS/MS, UPLC-MS/MS	[34]
		1-linoleoyl-2-linolenoyl-GPC (18:2/18:3)	C_44_H_84_NO_8_P	plasma	LC-MS/MS, UPLC-MS/MS	[34]
		1-myristoyl-2-palmitoyl-GPC (14:0/16:0)	C_38_H_76_NO_8_P	plasma	LC-MS/MS, UPLC-MS/MS	[34]
		1-oleoyl-2-docosahexaenoyl-GPC (18:1/22:6)	C_48_H_82_NO_8_P	plasma	LC-MS/MS, UPLC-MS/MS	[34]
		1-stearoyl-2-arachidonoyl-GPC (18:0/20:4)	C_46_H_84_NO_8_P	plasma	LC-MS/MS, UPLC-MS/MS	[34]
		Glycerophosphorylcholine (GPC)	C_8_H_20_NO_6_P	plasma	LC-MS/MS, UPLC-MS/MS	[34]
		Lysophosphatidylcholine	C_10_H_22_NO_7_P	plasma	GC-MS, LC-MS/MS, H-NMR	[55]
		Phosphatidylcholine	C_46_H_88_NO_8_P	plasma	GC-MS, LC-MS/MS, H-NMR	[55]
		Phosphatidylcholine diacyl C32:1, c36:5		plasma	UPLC-MS/MS, GC-MS	[63]
	down	**Sphingomyelin** (d18:1/19:0, d19:1/18:0), (d18:1/20:1, d18:2/20:0), (d18:1/22:1, d18:2/22:0, d16:1/24:1), (d18:2/18:1), (d18:2/21:0, d16:2/23:0)	C_47_H_93_N_2_O_6_P	plasma	GC-MS, LC-MS/MS, UPLC-MS/MS	[34,55,72]
		1-(1-enyl-stearoyl)-GPE (p-18:0)	C_23_H_48_NO_6_P	plasma	LC-MS/MS, UPLC-MS/MS	[34]
		1-linoleoyl-GPE (18:2)	C_23_H_44_NO_7_P	plasma	LC-MS/MS, UPLC-MS/MS	[34]
**Glycerolipids**	down	C18:0 cholesteryl ester	C_48_H_82_O_2_	plasma	UPLC-MS/MS	[60]
	up	C20:5 cholesteryl ester	C_20_H_32_O_2_	plasma	UPLC-MS/MS	[60]
	up	C34:1 phosphatidylcholine	C_40_H_80_NO_8_P	plasma	UPLC-MS/MS	[60]
**Steroids and steroid derivatives**	up	Cholesterol sulfate	C_27_H_46_O_4_S	feces	UPLC-QTOF-MS, UPLC-ESI-MS/MS	[26,27]
	down	Sulfolithocholic acid	C_24_H_40_O_6_S	feces	UPLC-QTOF-MS, UPLC-ESI-MS/MS	[26,27]
		Tetrahydrocortisol sulfate	C_21_H_33_NaO_8_S	plasma	LC-MS/MS, UPLC-MS/MS	[34]
	up	Deoxycholic acid	C_24_H_40_O_4_	feces	UPLC-QTOF-MS, UPLC-ESI-MS/MS	[26,27]
		Isoursodeoxycholic acid	C_24_H_40_O_4_	plasma	LC-MS/MS, UPLC-MS/MS	[34]
		Deoxycholic acid glucuronide	C_30_H_48_O_10_	plasma	LC-MS/MS, UPLC-MS/MS	[34]
		Glycodeoxycholate 3-sulfate	C_26_H_43_NO_8_S	plasma	LC-MS/MS, UPLC-MS/MS	[34]
		Dexydroepiandrosterone sulfate	C_19_H_28_O_5_S	plasma	GC-MS, LC-MS/MS, H-NMR	[55]
		4-androsten-3beta,17beta-diol disulfate	C_19_H_30_O_8_S_2_	plasma	UPLC-MS/MS, GC-MS	[63]
	up	5alpha-androstan-3alpha,17beta-diol monosulfate	C_19_H_31_O_5_S^−^	plasma	LC-MS/MS, UPLC-MS/MS	[34]
		5alpha-androstan-3beta,17beta-diol disulfate	C_19_H_32_O_8_S_2_	plasma	UPLC-MS/MS, GC-MS	[63]
	up	Androstenediol (3β,17β) monosulfate	C_19_H_28_O_6_S	plasma	UPLC-MS/MS	[34,72]
		Epiandrosterone sulfate	C_19_H_30_O_5_S	plasma	UPLC-MS/MS, GC-MS	[63]
	up	Andro steroid monosulfate	C_19_H_28_O_6_S	plasma	LC-MS/MS, UPLC-MS/MS	[34]
**Monoterpenoids**		1-methyl-4-(1-methyl-2-propenyl)-benzene	C_13_H_18_	urine	UPLC-QTOF-MS	[24]
	up	Valechlorin	C_22_H_31_ClO_8_	urine	UPLC-QTOF-MS	[25]
**Terpene glycosides**		16,17-dihydro-16a,17-dihydroxygibberellin a4 17-glucoside	C_25_H_36_O_12_	urine	UPLC-QTOF-MS	[24,25]

**Table 5:** Organic acids and derivatives affected by wine consumption.

	Effect of Wine Consumption	Name	Molecular Formula	Sample	Analytical Technique	Ref.
**Aliphatic**	down	Formic acid	CH_2_O_2_	urine	H-NMR	[50]
	up	Lactic acid	C_3_H_6_O_3_	urine	H-NMR	[44]
	up	**Tartaric acid**	C_4_H_6_O_6_	urine	LC–ESI-MS/MS, UHPLC-TOF MS, H-NMR	[25,41,42,44,45,47,65]
		Isobutyric acid	C_4_H_8_O_2_	feces	UPLC-ESI-MS/MS, SPME-GC-MS, H-NMR	[22,23,36]
		2-hydroxybutyric acid (AHD)	C_4_H_8_O_3_	plasma	UPLC-MS/MS, GC-MS	[63]
	down	2R,3R-dihydroxybutyric acid	C_4_H_8_O_4_	plasma	LC-MS/MS, UPLC-MS/MS	[34]
	up	Glutaric acid	C_5_H_8_O_4_	feces	UPLC-QTOF-MS, UPLC-ESI-MS/MS	[26,27]
	up	2-hydroxyglutaric acid	C_5_H_8_O_5_	urine, feces	UPLC-QTOF-MS, UPLC-ESI-MS/MS	[24,26,27]
		3-methyl-2-oxobutyrate	C_5_H_8_O_3_	plasma	UPLC-MS/MS, GC-MS	[63]
		Citric acid	C_6_H_8_O_7_	urine	UPLC-QTOF-MS	[24]
	up	Methylisocitric acid	C_7_H_10_O_7_	urine	UPLC-QTOF-MS	[25]
	up	Monoglyceride citrate	C_9_H_14_O_9_	urine	UPLC-QTOF-MS	[25]
	up	Gulonate	C_6_H_11_O_7_^−^	plasma	LC-MS/MS, UPLC-MS/MS	[34]
		4-methyl-2-oxopentanoate	C_6_H_10_O_3_	plasma	UPLC-MS/MS, GC-MS	[63]
	up	2-isopropyl-3-oxosuccinate	C_7_H_10_O_5_	urine	UPLC-QTOF-MS	[25]
	up	2-oxovaleric acid	C_5_H_8_O_3_	urine	UPLC-QTOF-MS	[24,25]
		γ-delta-dioxovaleric acid	C_5_H_6_O_4_	urine	UPLC-QTOF-MS	[24]
	up	3-methyl-2-oxovalerate	C_6_H_10_O_3_	urine, plasma	HPLC-MS/MS, H-NMR	[34,45,47]
	down	Dimethylguanidino valeric acid (DMGV)	C_8_H_15_N_3_O_3_	plasma	UPLC-MS/MS	[60]
	down	Tricarballylic acid	C_6_H_8_O_6_	feces	UPLC-QTOF-MS, UPLC-ESI-MS/MS	[26,27]
	down	3-indoxylsulfuric acid	C_8_H_7_NO_4_S	urine	GC-MS, LC-MS/MS, H-NMR	[55]
	up	(E)-2-propenyl [3-(2-propenylthio)-2-propenyl] sulfate	C_9_H_14_O_4_S_2_	urine	UPLC-QTOF-MS	[25]
**Benzene and substituted derivatives**		Benzoate	C_7_H_5_O_2_^−^	plasma	UPLC-MS/MS, GC-MS	[75]
	up	Benzoic acid	C_7_H_6_O_2_	feces	UPLC-ESI-MS/MS, UPLC-TOF-MS	[26,27]
		Sulfosalicylic acid	C_7_H_6_O_6_S	urine	UPLC-QTOF-MS	[24,25]
		Phenylacetic acid	C_8_H_8_O_2_	urine, feces	UPLC-MS/MS, UPLC-ESI-MS/MS	[22,23,46]
		2-hydroxyphenylacetic acid	C_8_H_8_O_3_	urine	UPLC−MS/MS	[46]
	up	**3-hydroxyphenylacetic acid**	C_8_H_8_O_3_	urine, plasma, feces	H-NMR, UPLC−MS/MS, GC-MS, LC-MS/MS, GC–TOF–MS, UPLC-ESI-MS/MS, UPLC-TOF-MS	[26,28,44,46,49,54,55,56]
	up	**4-hydroxyphenylacetic acid**	C_8_H_8_O_3_	urine	UPLC-ESI-MS-MS, H-NMR, GC-MS, H-NMR, GC–TOF–MS	[44,47,49,50,56,68]
		**3,4-dihydroxyphenylacetic acid**	C_8_H_8_O_4_	urine, plasma, feces	UPLC-ESI-MS/MS, GC-MS	[22,46,49,68]
		οrtho-hydroxyphenylacetic acid	C_8_H_8_O_3_	plasma	LC-MS/MS, UPLC-MS/MS	[34]
		N-formylanthranilic acid	C_8_H_7_NO_3_	plasma	LC-MS/MS, UPLC-MS/MS	[34]
	up	**Hippuric acid**	C_9_H_9_NO_3_	urine	GC-MS, H-NMR, GC–TOF–MS	[47,49,50,55,56]
		**3-hydroxyhippuric acid**	C_9_H_9_NO_4_	urine	GC-MS, GC–TOF–MS, LC-MS/MS	[49,55,56]
	up	**4-hydroxyhippuric acid**	C_9_H_9_NO_4_	urine, plasma	UPLC−MS/MS, ΝΜR, GC-MS, H-NMR, GC–TOF–MS, LC-MS/MS	[24,46,49,50,54,55,56]
		Phenylpropionic acid	C_9_H_10_O_2_	feces	UPLC-ESI-MS/MS	[22,55,56]
	up	3-phenylpropionic acid (hydrocinnamic acid)	C_9_H_10_O_2_	feces	UPLC-TOF-MS, UPLC-ESI-MS/MS	[26,28]

**Table 6:** Amino acids and derivatives affected by wine consumption.

Categorization	Effect of Wine Consumption	Name	Molecular Formula	Sample	Analytical Technique	Ref.
**Amino Acids**	down	**Alanine**	C_3_H_7_NO_2_	urine	H-NMR	[50]
**Alanine Derivatives**	up	N-acetyl-beta-alanine	C_5_H_9_NO_3_	plasma	LC-MS/MS, UPLC-MS/MS	[34]
	up	1-carboxyethylphenylalanine	C_12_H_15_NO_4_	plasma	LC-MS/MS, UPLC-MS/MS	[34]
**Amino Acids**	down	**Threonine**	C_4_H_9_NO_3_	urine, plasma	H-NMR, GC-MS, LC-MS/MS	[44,55]
**Amino Acids**	down	**Lysine**	C_6_H_14_N_2_O_2_	plasma	GC-MS, LC-MS/MS, H-NMR	[55]
**Lysine Derivatives**		Pipecolic acid	C_6_H_11_NO_2_	plasma	UPLC-MS/MS, GC-MS	[63]
**Amino Acids**	down	**Tyrosine**	C_9_H_11_NO_3_	plasma	GC-MS, LC-MS/MS, H-NMR	[55]
**Amino Acids**		L-alpha-aminobutyric acid	C_4_H_9_NO_2_	plasma	UPLC-MS/MS, GC-MS	[63]
**Amino Acids**	down	S-methylcysteine sulfoxide (kale anemia factor)	C_4_H_9_NO_3_S	plasma	LC-MS/MS, UPLC-MS/MS	[34]
**N-acyl-alpha amino acids**	down	Acetylcysteine	C_5_H_9_NO_3_S	urine	UPLC-QTOF-MS	[25]
**Amino Acids**	down	2-ketocaprylate	C_8_H_14_O_3_	plasma	LC-MS/MS, UPLC-MS/MS	[34]
**Amino Acids**	down	Alliin	C_6_H_11_NO_3_S	plasma	LC-MS/MS, UPLC-MS/MS	[34]
**Non-essential amino acids**	up	Carnitine	C_7_H_15_NO_3_	plasma	UPLC-MS/MS	[60]
**Uncommon amino acids**	up	gamma-Carboxyglutamic acid	C_6_H_9_NO_6_	plasma	LC-MS/MS, UPLC-MS/MS	[34]
**Alpha amino acids and derivatives**	down	Guanidinoacetate	C_3_H_7_N_3_O_2_	plasma	LC-MS/MS, UPLC-MS/MS	[34]
**Histidine derivatives**	up	1-methylhistidine	C_7_H_11_N_3_O_2_	urine	GC-MS, LC-MS/MS, H-NMR	[55]
**l-cysteine-s-conjugates**	up	Lanthionine	C_6_H_12_N_2_O_4_S	plasma	LC-MS/MS, UPLC-MS/MS	[34]
**Glutamic acid derivatives**	down	Glutamic acid gamma-methyl ester (PGMGT)	C_6_H_11_NO_4_	plasma	LC-MS/MS, UPLC-MS/MS	[34]
**N-acyl-alpha amino acids**	up	N-acetylglutamine	C_7_H_12_N_2_O_4_	plasma	LC-MS/MS, UPLC-MS/MS	[34]
**Tryptamine derivatives**	down	Serotonin	C_10_H_12_N_2_O	plasma	LC-MS/MS, UPLC-MS/MS	[34]
	down	N-methyltryptamine	C_11_H_14_N_2_	urine	UPLC-QTOF-MS	[25]
**Metabolites of the Tryptophan-Niacin catabolic pathway**		2-amino-3-carboxymuconic acid semialdehyde	C_7_H_7_NO_5_	urine	UPLC-QTOF-MS	[24]
**Reductive products of tryptophan**	up	Indole-3-propionic acid	C_11_H_11_NO_2_	plasma	LC-MS/MS, UPLC-MS/MS	[34]
**Tryptophan metabolites**	up	Indole-3-lactic-acid	C_11_H_11_NO_3_	urine	GC-MS, LC-MS/MS, H-NMR	[55]
**Indolyl carboxylic acids and derivatives**	up	Tryptophan 2-c-mannoside	C_17_H_22_N_2_O_7_	plasma	LC-MS/MS, UPLC-MS/MS	[34]
**N-trimethylated amino acids**	up	Betaine	C_5_H_11_NO_2_	urine	H-NMR	[44]
**Canavanine biosynthesis pathway**	up	O-ureidohomoserine	C_5_H_11_N_3_O_4_	urine	UPLC-QTOF-MS	[25]
**Valine and derivatives**	down	4-hydroxyvalsartan	C_24_H_29_N_5_O_4_	urine	UPLC-QTOF-MS	[25]
**Hormones**	down	Thyroxine	C_15_H_11_I_4_NO_4_	plasma	LC-MS/MS, UPLC-MS/MS	[34]
	up	3-methylcrotonylglycine	C_7_H_11_NO_3_	urine	UPLC-QTOF-MS	[25]
**Dipeptides**	up	Cysteinylglycine disulfide	C_8_H_15_N_3_O_5_S_2_	plasma	LC-MS/MS, UPLC-MS/MS	[34]
		Aspartyl-leucine/leucyl-aspartate	C_10_H_18_N_2_O	urine	UPLC-QTOF-MS	[24]
	down	Hydroxyprolyl-(iso)leucine	C_11_H_20_N_2_O_4_	urine	UPLC-QTOF-MS	[25]
	up	L-γ-glutamyl-l-(iso)leucine	C_11_H_20_N_2_O_5_	urine	UPLC-QTOF-MS	[25]
		Hypoglycin b	C_12_H_18_N_2_O_5_	urine	UPLC-QTOF-MS	[24]
	up	Phenylalanylaspartic acid	C_13_H_16_N_2_O_5_	urine	UPLC-QTOF-MS	[25]
**Hybrid peptides**	down	Bis-γ-glutamylcysteinylbis-β-alanine	C_22_H_36_N_6_O_12_S_2_	urine	UPLC-QTOF-MS	[25]
**Peptides**	down	Phytosulfokine a	C_33_H_46_N_6_O_16_S_2_	urine	UPLC-QTOF-MS	[25]

**Table 7:** Carbohydrates and carbohydrate conjugates affected by wine consumption.

	Effect of Wine Consumption	Name	Molecular Formula	Sample	Analytical Technique	Ref.
Polyols	up	Erythritol	C_4_H_10_O_4_	plasma	LC-MS/MS, UPLC-MS/MS	[34]
	up	Ribitol	C_5_H_12_O_5_	plasma	LC-MS/MS, UPLC-MS/MS	[34]
	up	Arabitol/Xylitol	C_5_H_12_O_5_	plasma	LC-MS/MS, UPLC-MS/MS	[34]
	up	Arabonate/Xylonate	C_5_H_10_CaO_6_	plasma	LC-MS/MS, UPLC-MS/MS	[34]
	up	L-Fucose	C_6_H_12_O_5_	urine	H-NMR	[44]
	up	**Scyllo-inositol/inositol**	C_6_H_12_O_6_	plasma	LC-MS, GC-MS, UHPLC-MS/MS	[60,62,63]
	up	Glucose	C_6_H_12_O_6_	urine	H-NMR	[44]
	up	Glucose-1-phosphate	C_6_H_13_O_9_P	urine	GC-MS, LC-MS/MS, H-NMR	[55]
	up	**Mannitol**	C_6_H_14_O_6_	urine	H-NMR	[44,45,47]
	up	Sedoheptulose	C_7_H_14_O_7_	plasma	LC-MS/MS, UPLC-MS/MS	[34]
	up	Sucrose	C_12_H_22_O_11_	urine	GC-MS, LC-MS/MS, H-NMR	[55]
Alkylglucosinolates	up	Glucosinalbin	C_14_H_18_NO_10_S_2_	urine	UPLC-QTOF-MS	[25]
		3-methylbutyl glucosinolate	C_12_H_23_NO_9_S_2_	urine	UPLC-QTOF-MS	[24]
n-acylneuraminic acids	down	N-Acetylneuraminate (sialic acid)	C_11_H_19_NO_9_	plasma	LC-MS/MS, UPLC-MS/MS	[34]

**Table 8:** Vitamins and energy-related molecules affected by wine consumption.

	Effect of Wine Consumption	Name	Molecular Formula	Sample	Analytical Technique	Ref.
Cofactors and Vitamins	up	**Nicotinic acid** (vitamin B3)	C_6_H_5_NO_2_	urine	GC-MS, LC-MS/MS, H-NMR	[55]
	up	Pantoic acid	C_6_H_12_O_4_	plasma	LC-MS/MS, UPLC-MS/MS	[34]
	up	Isocitric lactone	C_6_H_6_O_6_	plasma	LC-MS/MS, UPLC-MS/MS	[34]
	down	Phosphate	O_4_P-_3_	plasma	LC-MS/MS, UPLC-MS/MS	[34]
	down	Cytidine triphosphate	C_9_H_16_N_3_O_14_P_3_	urine	UPLC-QTOF-MS	[25]

**Table 9:** Other Organic compounds metabolites affected by wine consumption.

		Effect of Wine Consumption	Name	Molecular Formula	Sample	Analytical Technique	Ref.
**Organic compounds (Aliphatic)**	**Organic oxygen compounds**	up	Methanol	CH_4_O	urine	H-NMR	[44]
			**Ethanol**	C_2_H_6_O	urine	H-NMR	[45,47]
		up	Ethyl hydrogen sulfate	C_2_H_6_O_4_S	urine, plasma	UHPLC-MS/MS	[25,40]
		up	**Ethyl glucuronide**	C_8_H_14_O_7_	plasma	UPLC-MS/MS, H-NMR	[45,59,72]
		up	Ethyl α-glucopyranoside	C_8_H_16_O_6_	plasma	UPLC-MS/MS	[59]
			Ethyl alpha-glucopyranoside	C_15_H_20_O_10_	plasma	LC-MS/MS, UPLC-MS/MS	[34]
		up	Dimethylamine (DMA)	C_2_H_7_N	urine	H-NMR	[44]
			2,3-butanediol	C_4_H_10_O_2_	urine	H-NMR	[45]
		up	2,3-pentanedione	C_5_H_8_O_2_	feces	UPLC-QTOF-MS, UPLC-ESI-MS/MS	[26,27]
	**Organosulfur compounds**	up	Ethyl 1-(ethylthio)propyl disulfide	C_7_H_16_S_3_	urine	UPLC-QTOF-MS	[25]
		down	Ajoene (2-propenyl-3-(2-propenylsulfinyl)-1-propenyl disulfide)	C_9_H_14_OS_3_	urine	UPLC-QTOF-MS	[25]
	**Organonitrogen compounds**	down	Linoleoyl ethanolamide	C_20_H_37_NO_2_	plasma	LC-MS/MS, UPLC-MS/MS	[34]
**Organic compounds (Cyclic)**	**Alcaloids**		Trigonelline	C_7_H_7_NO_2_	urine	H-NMR, GC-TOF-MS	[49]
		up	Piperine	C_17_H_19_NO_3_	plasma	LC-MS, GC-MS	[60,63]
	**Organoheterocyclic compounds**		2-Furanmethanol	C_5_H_6_O_2_	urine	UPLC-QTOF-MS	[24]
		up	Dihydropteridine	C_6_H_6_N_4_	urine	UPLC-QTOF-MS	[25]
		up	Ethyl maltol	C_7_H_8_O_3_	urine	UPLC-QTOF-MS	[25]
		down	5-hydroxyindole	C_8_H_7_NO	urine	UPLC-QTOF-MS	[25]
			(R)-2,3-dihydro-3,5-dihydroxy-2-oxo-3-indoleacetic acid	C_10_ H_9_NO_5_	urine	UPLC-QTOF-MS	[24]
		up	1-(2,3-dihydro-1h-pyrrolizin-5-yl)-2-propen-1-one	C_10_H_11_NO	urine	UPLC-QTOF-MS	[25]
		up	4-[(2,4-dihydroxyphenyl)azo] benzenesulfonic acid	C_12_H_10_N_2_O_5_S	urine	UPLC-QTOF-MS	[25]
		down	5-(1-propynyl)-5′-vinyl-2,2′-bithiophene	C_13_H_10_S_2_	urine	UPLC-QTOF-MS	[25]
			5-ethynyl-5′-(1-propynyl)-2,2′-bithiophene	C_13_H_8_S_2_	urine	UPLC-QTOF-MS	[24]
		up	Stercobilin	C_33_H_46_N_4_O_6_	feces	UPLC-QTOF-MS, UPLC-ESI-MS/MS	[26,27]
		up	Urobilinogen	C_33_H_44_N_4_O_6_	feces	UPLC-QTOF-MS, UPLC-ESI-MS/MS	[26,27]
	**Benzene and supstituted derivatives**	up	p-chlorobenzene sulfonyl urea	C_7_H_7_ClN_2_O_3_S	urine	UPLC-QTOF-MS	[25]
		down	p-cresol sulfate	C_7_H_8_O_4_S	urine	GC-MS, LC-MS/MS, H-NMR	[55]
		up	**4-hydroxy-5-(phenyl)-valeric acid**	C_11_H_14_O_3_	feces	UPLC-QTOF-MS, UPLC-ESI-MS/MS	[22,26,27,28,35]
		up	4-hydroxy-5-(phenyl)-valeric acid-o-sulfate	C_11_H_14_O_6_S	urine	UPLC-QTOF-MS	[25]
		up	4-hydroxy-5-(phenyl)-valeric acid-o-glucuronide	C_17_H_22_O_9_	urine	UPLC-QTOF-MS	[25]
		up	Salicylate glucuronide	C_13_H_14_O_9_	urine	UPLC-QTOF-MS	[25]
		up	Dibenzyl disulfide	C_14_H_14_S_2_	urine	UPLC-QTOF-MS	[25]
		down	Cinnamyl cinnamate	C_18_H_16_O_2_	urine	UPLC-QTOF-MS	[25]
			Flavidulol c	C_34_H_42_O_4_	urine	UPLC-QTOF-MS	[25]
	**Purines and purine derivatives**	down	Xanthine	C_5_H_4_N_4_O_2_	feces	UPLC-QTOF-MS, UPLC-ESI-MS/MS	[26,27]
		up	Uric acid	C_5_H_4_N_4_O_3_	plasma	GC-MS	[38]
			3-methylxanthine	C_6_H_6_N_4_O_2_	plasma	LC-MS/MS, UPLC-MS/MS	[34]
		up	1,3-dimethyluric acid	C_7_H_8_N_4_O_3_	plasma	LC-MS/MS, UPLC-MS/MS	[34]
	**Pyrimidines and pyrimidine derivatives**		Theophylline	C_7_H_8_N_4_O_2_	plasma	UPLC-MS/MS, GC-MS	[63]
		up	Caffeine	C_8_H_10_N_4_O_2_	plasma	UPLC-MS/MS	[60]
		up	5-acetylamino-6-formylamino-3-methyluracil	C_8_H_10_N_4_O_4_	urine	UPLC-QTOF-MS	[25]
		up	Nicotine	C_10_H_14_N_2_	urine	UPLC-MS, UPLC-MS/MS, GC-MS	[69]
		up	Cotinine	C_10_H_12_N_2_O	plasma	UPLC-MS/MS	[60]
	**Pyrimidine nucleosides**		Deoxyuridine	C_9_H_12_N_2_O_5_	plasma	LC-MS/MS, UPLC-MS/MS	[34]
	**Pyrrolidines**	up	Acisoga [n-(3-acetamidopropyl)pyrrolidin-2-one]	C_9_H_16_N_2_O_2_	plasma	LC-MS/MS, UPLC-MS/MS	[34]

## Supplementary Materials

The following are available online at https://www.mdpi.com/article/10.3390/molecules28227616/s1, Table S1: Interventional and Observational Clinical Studies in Wine Metabolomics Research.
