# Supplementary material for: Exploring Human Metabolome after Wine Intake—A Review
Source: Molecules. 2023 Nov 15;28(22):7616. doi: 10.3390/molecules28227616 (PMC10673339; doi:10.3390/molecules28227616)
Supplement: Supplementary file 1 [file molecules-28-07616-s001.zip › molecules-2670501-supplementary.pdf]

**Supplementary Table S1.** Interventional and Observational Clinical Studies in Wine Metabolomics Research

| Goal of the study | Study type | Intervention period | Number of Participants (gender) | Sexe | Health of participants | Control Group | Wine (mL)/day | Type of wine (variety of wine) | Matrix | Metabolomic Analysis | Analytical Technique | Sample Preparation | Statistical Analysis | Ref. |
|---|---|---|---|---|---|---|---|---|---|---|---|---|---|---|
| Association of previously-defined metabotypes with specific microbiological patterns and its response to a moderate wine intake, by combining metataxonomic and metabolomics data. | parallel interventional | 4 weeks | 19 | male, female | healthy | abstention (5) | 250 | RW (not mentioned) | feces | Targeted | UPLC-ESI-MS/MS (-), SPME-GCMS | DVB/CAR/PDMS SPME | Shapiro–Wilk test, Levene test, One-way ANOVA, Tukey's test, Student's t-test | [22] |
| Assess the changes in Fecal Water cytotoxicity after a wine intervention and establish a possible relationship between FW cytotoxicity and fecal microbial-derived metabolites | parallel interventional | 4 weeks | 8 | male, female | healthy | abstention (4) | 250 | RW (Pinot Noir) | feces | Targeted | UPLC-ESI-MS/MS (-), SPME-GCMS | **phenolic metabolites**: dilution with NaCl and again with ACN/water **short-chain fatty acids:** SPE | Student's t-test | [23] |

| | | | | | | | | | | | | | | |
|---|---|---|---|---|---|---|---|---|---|---|---|---|---|---|
| Evaluate the potential biological effects of moderate wine consumption on human microbiota and the fecal metabolome of healthy subjects. | parallel interventional | 4 weeks | 41 | male, female | healthy | abstention (8) | 250 | RW (Pinot Noir) | feces | Targeted and Non targeted | UPLC-ESI-MS/MS (-), UPLC-TOF/MS (-) | dilution with NaCl and again with ACN | Shapiro-Wilk test, Student's t-test, Mann-Whitney test, Wilcoxon matched-pairs test, PCA | [26] |
| Assess the changes in the profile of phenolic metabolites in feces after wine consumption, and stratify the population based on their ability to metabolize wine polyphenols. | parallel interventional | 4 weeks | 41 | male, female | healthy | | 250 | RW (Pinot Noir) | feces | Targeted | UPLC-ESI-MS/MS (-) | dilution with NaCl and again with ACN | Student's t-test, Mann-Whitney test, Wilcoxon matched-pairs test, one-way ANOVA, Shapiro-Wilk test | [28] |
| Delve deeper into biological effects exerted by phenolic compounds by studying the metabolomic fingerprint of faecal samples. | parallel interventional | 4 weeks | 41 | male, female | healthy | abstention (8) | 250 | RW (Pinot Noir) | feces | Non targeted | UPLC-TOF-MS (-) | dilution (x2) with NaCl, filtered with polyvinylidene difluoride (PVDF) membrane | Shapiro-Wilk test, Student's t-test, Wilcoxon matched-pairs test, PCA | [27] |

| | | | | | | | | | | | | | | |
|---|---|---|---|---|---|---|---|---|---|---|---|---|---|---|
| Examine the relationship between the plasma levels of polyphenols and the antioxidant activity of red and white wine | parallel interventional | 15 days | 20 | male, female | healthy | Abstention (10) | 300 | RW, WW (not mentioned) | plasma | Targeted | HPLC-ESA | hydrolysis with glucuronidase/sulfatase, acetate buffer (pH 5.0), LLE with ethyl acetate | one-way ANOVA, non-parametric tests (Wilcoxon test, Kolmogorov-Smirnov test), Levene test, MANOVA, Bonferroni test, correlation analysis using Pearson's test | [29] |
| Verify the bioavailability of free and glucuronide t-RESV and association with or without a meal and its composition | parallel interventional | 1 day | 25 (10,5, 5 all m) | m | healthy | - | 300, 600, 600 | RW (Lambrusco, Cabernet Franc, Agliatico) | serum | Targeted | HPLC-UV-Vis (+), HPLC-MS (-), HPLC-MS/MS (-) | LLE with ethyl acetate | - | [30] |
| Examine the changes in the overall urinary metabolome after moderate wine consumption. | parallel interventional | 4 weeks | 41 | male, female | healthy | abstention (8) | 250 | RW (Pinot Noir) | urine | Non targeted | UPLC-TOF-MS (-) | centrifugation and direct analysis | PCA, Student’s t-test, OPLS-DA | [25] |

| | | | | | | | | | | | | | | |
|---|---|---|---|---|---|---|---|---|---|---|---|---|---|---|
| Investigate the differential responsiveness of healthy subjects to moderate wine consumption and explore the differences in their metabotypes. | parallel interventional | 4 weeks | 41 | male, female | healthy | abstention (8) | 250 | RW (Pinot Noir) | urine | Non targeted | UPLC-QTOF-MS (-) | centrifugation and direct analysis | PCA, HCA, one-way ANOVA, OPLS-DA | [24] |
| Study the pharmacokinetics of resveratrol metabolites after moderate RW consumption and grape extract tablets | parallel interventional | 1 day | 10 | male | healthy | grape EXT tablets (3) | 375 | RW (Merlot) | urine, plasma | Targeted | LC–ESI–MS/MS (-) | HLB SPE | Mann–Whitney U test, Wilcoxon test | [31] |
| Investigate the profile of microbial phenolic metabolites obtained from red wine intake and compare it to samples taken after consuming dealcoholized red wine and gin. | crossover interventional | 20 days | 8 | male, female | healthy | | 272, 100 (gin) | RW, DRW, gin (Merlot) | feces | Targeted | UPLC-ESI-MS/MS (-) | dilution with NaCl and again with ACN | Student's t-test, nonparametric Wilcoxon matched-pairs test, PCA | [35] |
| Investigate the H-NMR applicability on the fecal metabolomics to dietary human intervention trials. | crossover interventional | 4 weeks | 53 | male, female | mildly hypertensive | | | capsules with mix of RW and GJ EXT | feces | Targeted | H-NMR | alkalized with NaOH and acidified with formic acid, LLE with $D_2O$, $CD_3OD$ both containing TSP | Student's t-test, PCA, PLS-DA | [36] |

| | | | | | | | | | | | | | | |
|---|---|---|---|---|---|---|---|---|---|---|---|---|---|---|
| Impact of red wine in men with heart coronary disease on plasma metabolome, gut microbiota, and trimethylamine N-oxide (TMAO) | crossover interventional | 3 weeks | 42 | male | CAD | - | 250 | RW (Merlot) | plasma | Non targeted | LC-MS/MS, UPLC-ESI-MS/MS (±) | PPT with MeOH | Shapiro-Wilk test, Student's t-test, paired Wilcoxon test, Spearman rank correlation test, Bonferroni test | [34] |
| Create a sensitive, precise, and selective analytical method for the identification and quantification of resveratrol metabolites in human low-density lipoprotein (LDL) following moderate red wine intake | crossover interventional | 1 day | 11 | male | healthy | | 250 | RW (Merlot) | plasma | Targeted | LC-ESI-MS/MS (±) | 3 different methods tested [HLB SPE (selected), PPT with acidified MeOH, LLE with ethyl acetate] | - | [37] |
| Determine the detectability of certain phenolic acids in plasma after red wine consumption and assess the potential effect on serum and LDL oxidation ex vivo. | crossover interventional | 1 day | 12 | male | healthy | | | RW, DRW, Phenol-stripped RW, water (Cabernet Shiraz) | plasma | Targeted | GC-MS | lyophilization, acetate buffer (pH 4.5), hydrolysis with b-glucuronidase, sulfatase, b-glucosidase, LLE with ethyl acetate, | ANOVA, general linear modeling | [38] |

| | | | | | | | | | | | | | |
|---|---|---|---|---|---|---|---|---|---|---|---|---|---|
| | | | | | | | | | | | derivatization with BSTFA | | |
| Evaluate changes in plasma (+)-catechin concentrations after a single, moderate serving of dealcoholized red wine reconstituted with either water or water and alcohol | crossover interventional | 1 day | 9 | male, female | healthy | 120 | water, DRW, water and alcohol (ARW) (Cabernet sauvignon ) | plasma | Targeted | GC-MS | hydrolysis with b-glucuronidase, arylsulfatase, LLE with methylene chloride/water and ethyl acetate, derivatization with BSTFA | Student's t test, Wilcoxon signed-rank test, Fisher's exact test | [39] |
| Determine the levels of catechin and its metabolites in plasma after the consumption of red wine and de-alcoholized red wine. | crossover interventional | 1 day | 9 | male, female | not mentio ned | 120 | RW, DRW (Cabernet Sauvignon ) | plasma | Targeted | GC-MS | hydrolysis with β-glucuronidase, sulfatase, LLE with methylene chloride/water and ethyl acetate, derivatization with BSTFA | Least-squares nonlinear regression, ANOVA, Student's t-test | [33] |
| Optimize a targeted metabolomics method for the analysis of diet-related metabolites in blood samples, ommiting an enzymatic hydrolysis step of phase II metabolites. | Crossover interventional | 4 weeks | 10 | male, female | healthy | 270 | RW (not mentioned ) | plasma | Targeted | UHPLC-MS/MS (±) | 3 different methods tested [PPT with ACN/formic acid/ammonium formate, HLB SPE, hybrid PPT] | Student's t-test | [40] |

| | | | | | | | | | | | | | |
|---|---|---|---|---|---|---|---|---|---|---|---|---|---|
| Evaluate tartaric acid as a possible biomarker of wine intake. | crossover interventional | 1 day | 21 | male | healthy | 100, 200, 300 | RW (Tempranillo (85 %), Graciano and Garnacha Tinta (15%)) | urine | Targeted | LC–ESI-MS/MS (-) | dilution with formic acid/water | omnibus K2 D'Agostino-Pearson test, Shapiro-Wilk test, non-parametric Mann-Whitney U test, Wilcoxon test, Clopper-Pearson exact binomial method. | [41] |
| Divide a population into phenotypic groups based on biochemical features - utilize H-NMR-based urinary metabolomics to examine the differed metabolic reactions following red wine polyphenol intake. | crossover interventional | 4 weeks | 57 | male, female | T2D or ≥3 CHD risk factors | | DRW (Merlot) | urine | Non targeted | H-NMR | Mixed with TSP, $NaN_3$, $KH_2PO_4$ in $D_2O$ - buffer KOD (pH=7) | OSC-PLS-DA | [44] |
| Evaluate the effectiveness of wine intake biomarkers in a wine intervention | crossover interventional | 4 weeks | 56 | male, female | T2D or ≥3 CHD | 272, 100 (gin) | RW, DRW, gin (not | urine | Non targeted | H-NMR | Mixed with TSP, $NaN_3$, $KH_2PO_4$ in $D_2O$ | Mann-Whitney U test, Mann- | [45] |

| | | | | | | | | | | | | | |
|---|---|---|---|---|---|---|---|---|---|---|---|---|---|
| study by using an NMR metabolomic approach. | | | | | risk factors | | mentioned ) | | | | | Whitney test, logistic regression model | |
| Determine the most comprehensive urinary metabolomic fingerprinting of phenolics and microbial-derived phenolic acids in males at high cardiovascular risk after regular consumption of dealcoholized red wine. | crossover interventional | 4 weeks | 36 | male | healthy | 272 | DRW (Merlot) | urine | Targeted | UPLC–MS /MS (-) | MCX SPE | Student’s t-test | [46] |
| Identify urinary metabolomic differences with H-NMR based metabolomic approach of moderate wine intake | crossover interventional | 4 weeks | 61 | male | T2D or ≥3 CHD risk factors | 272, 100 (gin) | RW, DRW, gin (Merlot) | urine | Non targeted | H-NMR | Mixed with TSP, $NaN_3$, $KH_2PO_4$ in $D_2O$ - buffer KOD (pH=7) | ANOVA test, Fisher's LSD test | [47] |
| Develop a sensitive and optimized analytical assay in order to more accurately quantify all known resveratrol metabolites | crossover interventional | 4 weeks | 10 | male | T2D or ≥3 CHD risk factors | 272 | RW (Merlot), DRW | urine | Targeted | UPLC–MS/MS (-) | HLB SPE | Kolmogorov test, Levene test, nonparametric Friedman test, paired Wilcoxon test | [48] |

| | | | | | | | | | | | | | |
|---|---|---|---|---|---|---|---|---|---|---|---|---|---|
| Investigate urinary excretion of microbial-origin phenolic acids after grape juice and wine extracts intake | crossover interventional | 4 weeks | 58 | male, female | mildly hypertensive | | capsules 2:1 polyphenol-rich mix of RW and GJ EXTs, capsules with GJ EXT | urine | **GC-TOF-MS**: target, H-NMR: Non targeted | H-NMR, GC-TOF-MS | **H-NRM**: phosphate buffer (pH 7, TSP) **GC-MS:** hydrolysis with β-glucuronidase, LLE with ethyl acetate, derivatization with BSTFA/trimethylchlorosilane | Wilcoxon test, ML-PLS-DA | [49] |
| Develop a fast, simple, and environmentally friendly method for analyzing major wine organic acids in human urine using LC−ESI-MS/MS | crossover interventional | 1 day | 5 | male | healthy | 200 | RW (Tempranillo) | urine | Targeted | LC−ESI-MS/MS (-) | dilution with water/formic acid | Student's t-test | [42] |
| Investigate the urinary excretion of catechin and its metabolites in human subjects following the intake of red wine and dealcoholized red wine. | crossover interventional | 1 day | 9 | male, female | healthy | 120 | RW, DRW (Cabernet Sauvignon) | urine | Targeted | GC-MS | hydrolysis with β-d-glucuronidase, sulfatase, LLE with ethyl acetate, derivatization with BSTFA | Student's t-test | [32] |
| Compare the alterations in plasma malvidin-3-glucoside and its urinary excretion after the ingestion of red wine, dealcoholized red wine, and red grape juice. | crossover interventional | 1 day | 6 | male | healthy | 500 | RW, DRW, GJ (Lemberger) | urine | Targeted | HPLC-UV-Vis (+) | C18 SPE | ANOVA, Fischer's test, linear regression analysis | [43] |

| | | | | | | | | | | | | | | | |
|---|---|---|---|---|---|---|---|---|---|---|---|---|---|---|---|
| 1) Compare multilevel PLS-DA against the ordinary PLS-DA approach, and 2) select and analyze candidate biomarkers after treatment effect. | crossover interventional | 4 weeks | 29 | male, female | mildly hypertensive | | | | capsules with mix of RW EXT and GJ EXT | urine | Non targeted | H-NMR | buffer phosphate and sodium salt (TSP)) (pH 3) | PLS-DA | [50] |
| Assess the impact of nano-encapsulation of a phenol extract on human plasma pharmacokinetic parameters, examine phenolic metabolite in urine clearance and identify new metabolites. | crossover interventional | 1 day | 12 | male, female | healthy | | 272, 100 (gin) | DRW with EXT, DRW with encapsulated EXT, gin | urine, plasma | Targeted | UPLC-ESI-MS/MS (±) | HLB SPE | ANOVA, Tukey's test | [51] |
| Develop a sensitive method about c-RESV, t-RESV and dihydroresveratrol in urine and plasma after red wine, grape juice and grape extracts (prepared as tablets) ingestion. | crossover interventional | 1 day | 11 | male | healthy | | 250, 1000 (GJ), 10 tablets | RW, GJ, capsules with RW EXT (not mentioned) | urine, plasma | Targeted | GC-MS | **urine**: dilution with water, **plasma/urine**: acetate buffer (pH 5.2), hydrolysis with β-glucuronidase, LLE with ACN/ethyl acetate (in urine a solution of NaCl were added before extraction), derivatised with MSTFA: $NH_4I$: 2- | least-square regression analysis | [52] |

| | | | | | | | | | | | | | |
|---|---|---|---|---|---|---|---|---|---|---|---|---|---|
| | | | | | | | | | | | mercaptoethanol reaction mixture (ammonium iodide and 2-mercaptoethanol per litter of MSTFA) | | |
| Discover and evaluate food intake biomarkers in urine and plasma phenolic and microbial profile by targeted metabolomics after wine intervention | crossover interventional | 4 weeks | 36 | male | T2D or ≥3 of the CVRFs | 272, 100 (gin) | RW, DRW (Merlot), gin | urine, plasma | Targeted | UPLC-ESI-MS/MS (-) | HLB SPE | PCA, HCA, ANOVA, Bonferroni test, Binary stepwise logistic regression analysis | [54] |
| Study metabolic effects of red wine and grape polyphenols in plasma and urine from healthy male adults consuming a mix of red wine and grape juice extracts. | crossover interventional | 5 days | 35 | male | healthy | 630 | capsules 2:1 polyphenol-rich mix of RW and GJ EXTs | urine, plasma | **H-NMR:** target**,** **GC-MS, LC-MS/MS:** target and Non targeted | GC-MS, LC-MS/MS (±), H-NMR | **plasma:** PPT with ACN, LLE with water/ethanol/dichloromethane. **urine:** dilution **H-NMR urine**: see (van Dorsten et al., 2010) **GC-MS:** fatty acid esterification, derivatization | Mann-Whitney U test, Wilcoxon test | [55] |

| | | | | | | | | | | | | | |
|---|---|---|---|---|---|---|---|---|---|---|---|---|---|
| | | | | | | | | | | | with O-methylhydroxyamine hydrochloride, MSTFA | | |
| Assess the bioavailability and bioactivity of anthocyanins of red grape juice versus red wine intake. | crossover interventional | 1 day | 9 | male, female | healthy | 400 | RW, GJ (Lemberger) | urine, plasma | Targeted | HPLC-UV-Vis (+) | C18 SPE | Shapiro-Wilk test, Student's t-test | [53] |
| Develop a new method for measuring catechin, quercetin, and resveratrol, as well as their conjugates, in plasma and urine. | crossover interventional | 1 day | 10 | not mentioned | not mentioned | | WW, GJ, vegetable cocktail (Lindemans Chardonnay) | urine, serum | Targeted | GC–MS | LLE with ethyl acetate, derivatization with BSTFA | Pearson test | [57] |
| Evaluate resveratrol metabolites as potential biomarkers of wine consumption, in humans after moderate consumption of sparkling, white, or red wines. | crossover interventional | 4 weeks | 52 | male, female | healthy | 200 (RW, WW), 300 (sparkling wine), 100 (gin) | RW, WW, gin (not mentioned) | urine, serum | Targeted | LC-MS/MS (-) | HLB SPE | Kolmogorov test, Levene test, Wilcoxon test, Student's t-test, ANOVA | [58] |

| | | | | | | | | | | | | | |
|---|---|---|---|---|---|---|---|---|---|---|---|---|---|
| Develop a GC–MS-based method for the profiling of phenolic microbial fermentation products in urine, plasma, and fecal water. | Crossover interventional | 4 weeks | 26 | male, female | not mentioned | | capsules with mix of RW EXT and GJ EXT | urine, plasma, feces | Non targeted | GC–TOF–MS | **urine, plasma:** hydrolysis with β-d-glucuronidase, acidification with HCl, LLE with ethyl acetate, derivatization: BSTFA/TMCS | PCA, OPLS-DA | [56] |
| Assess the relationships between short-term and long-term dietary intake of resveratrol and wine and urinary resveratrol excretion in a European population. | observational | | 475 | male, female | general population | | | urine | Targeted | UHPLC-ESI-MS/MS (+) | hydrolysis with β-glucuronidase, sulfatase, LLE with ethyl acetate | one-way ANOVA, Spearman's rank correlations, partial Spearman's correlations | [66] |
| Determine whether resveratrol could serve as a reliable biomarker for accurately measuring wine consumption in a specific population, compared to | observational | | 25 | not mentioned | healthy | | | plasma | Not targeted | HPLC-ESI-MS/MS (± tested but – preferred) | PPT with acetic acid, HLB SPE | not used a statistical analysis test rather than | [61] |

| | | | | | | | | | | |
|---|---|---|---|---|---|---|---|---|---|---|
| self-reported dietary assessments. | | | | | | | | | statistical parameters | |
| Develop a novel algorithm to identify urinary polyphenol metabolite patterns explaining the intake of polyphenol-rich foods and compare their performance with individual biomarkers in assessing dietary intake within the EPIC study. | observational | 475 | male, female | general popula tion | urine | Targeted | UPLC-ESI-MS-MS (+) | hydrolysis with β-glucuronidase, sulfatase LLE with ethyl acetate | expectation - maximizati on (EM) algorithm, GLMs, RRR-VIP method, reduced rank regression, LASSO regression, RRR analysis, internal two-fold cross-validation | [67] |
| Compare polyphenol urine excretion in individuals from different European countries with a wide range of polyphenol intakes and lifestyle factors. | observational | 1386 | male, female | general popula tion | urine | Targeted | UPLC-ESI-MS/MS | hydrolysis with β-glucuronidase, sulfatase LLE with ethyl acetate | PC-PR2, one-way ANOVA | [68] |

| | | | | | | | | | | |
|---|---|---|---|---|---|---|---|---|---|---|
| Discover a set of metabolites to discriminate red wine consumers. | observational | 1157 | male, female | T2D or ≥3 major CVRFs | plasma | Non targeted | UPLC-MS/MS (-) | **amino acids and other polar metabolites**: LLE with ACN /MeOH/formic acid, **lipids:** LLE with isopropanol | cross-validation | [60] |
| Identify and evaluate metabolites reproducibility that are biomarkers of usual dietary intake. | observational | 502 | male, female | healthy | plasma | Non targeted | UPLC-ESI-MS/MS (±) | PPT with MeOH | 2-sided statistical tests (chi-square test, Mann-Whitney U Test), FDRs, LASSO regression | [62] |
| Evaluate the relationship between more than 100 food groups/items and 1141 metabolites measured six months apart in racial and ethnically diverse men and women. | observational | 671 | male, female | general popula tion | plasma | Targeted | UPLC-ESI-MS/MS (±) | PPT with MeOH | Pearson's partial correlation | [59] |
| Discover and assess the reliability of new biomarkers that can indicate habitual food intake | observational | 3559 | female | general popula tion | plasma, serum | Targeted and Non targeted | UPLC-MS/MS (±), GC-MS | **UPLC**: PPT with MeOH, **GC**: PPT with MeOH, derivatization: BSTF | linear regression analysis | [63] |

| | | | | | | | | | | | | |
|---|---|---|---|---|---|---|---|---|---|---|---|---|
| Verify previously reported food-metabolite connections and uncover novel metabolomics-based biomarkers. | observational | | 849 | male, female | general popula tion | | serum | Non targeted | UPLC-ESI-MS/MS (-) | LLE with MeOH | Student's t-tests, chi-square test | [64] |
| Discover potential food biomarkers and assess their accuracy in predicting. | observational | | 1369 | female | healthy postme nopaus al women | | serum, urine | Non targeted | UPLC-ESI-MS/MS (±) | PPT with MeOH | Pearson's partial correlation, linear Support Vector Machine multivariat e classificati on model | [72] |
| Establish a correlation between urinary tartaric acid as a biomarker of wine consumption and cardiovascular risk factors in postmenopausal women at risk of developing cardiovascular disease. | observational | | 222 | male, female | T2D or ≥3 major CVRFs | | urine | Targeted | LC–ESI-MS/MS (-) | dilution with water/formic acid | Shapiro-Wilk test, multiple adjusted linear regression models | [65] |
| Validate urinary resveratrol metabolites as a biomarker of wine consumption in PREDIMED trial | observational | | 1000 | male, female | T2D or ≥3 CHD risk factors | | urine | Targeted | UPLC-MS/MS (-) | HLB SPE | chi-square tests, Kolmogoro v test, Levene test, one way | [71] |

| | | | | | | | | | | |
|---|---|---|---|---|---|---|---|---|---|---|
| Compare urine and serum metabolite profiles of habitual diet | observational | 253 | male, female | new or recurrent colorectal adenoma cases and adenoma-free controls | urine | Non targeted | UPLC-MS, UPLC-ESI-MS/MS (±), GC-MS | direct analysis | ANOVA, Mann-Whitney test Wilcoxon's signed rank test, chi-square test, Partial Pearson correlation, FDR calculation (Benjamini-Hochberg procedure), LASSO regression | [69] |
| Identify novel biomarkers of intake for a selection of polyphenol containing foods by metabolomics approach | observational | 481 | male, female | general population | urine | Non targeted | UPLC-QTOF-MS (-) | dilution with water | OPLS-DA, HCA | [70] |

T2D: type 2 diabetes, CAD: coronary artery disease, CHD: coronary heart disease, CVRFs: cardiovascular risk factors PPT: protein precipitation, DVB/CAR/PDMS: Divinylbenzene/Carboxen/Polydimethylsiloxane, ACN: acetonitrile, MeOH: methanol, BSTFA: N,O-bis (trimethylsilyl)trifluoroacetamide, TSP: 3-(trimethylsilyl)-proprionate-2,2,3,3-$d_4$, $NaN_3$: sodium azide, $D_2O$: deuterium water, KOD: potassium deuteroxide, $CD_3OD$: deuterated methanol, LC: liquid chromatography, MS/MS: tandem mass spectrometry, UPLC: ultra-high performance liquid chromatography, SPME: solid phase microextraction, DDA: data dependent acquisition, MRM: multiple reaction monitoring, PCA: Principal component analysis, ANOVA: Analysis of Variance, MANOVA: Multivariate analysis of variance, PLS-DA: Partial Least Squares Discriminant Analysis OPLS-DA: Orthogonal Partial Least Squares Discriminant Analysis, OSC-PLS-DA: Orthogonal Signal Correction-Partial Least Squares Discriminant Analysis, HCA: Hierarchical Clustering Analysis, PC-PR2: principal component partial R-square analysis, GLMs: general linear models, FDR: false discovery rate, LASSO: least absolute shrinkage and selection operator, LSD: Least Significant Difference, nm: not mentioned
